# Unusual Defect-Related Room-Temperature Emission from WS_2_ Monolayers Synthesized through a Potassium-Based Precursor

**DOI:** 10.1021/acsomega.3c03476

**Published:** 2023-10-03

**Authors:** Peter Walke, Reelika Kaupmees, Maarja Grossberg-Kuusk, Jüri Krustok

**Affiliations:** Department of Materials and Environmental Technology, Tallinn University of Technology, Ehitajate tee 5, 19086 Tallinn, Estonia

## Abstract

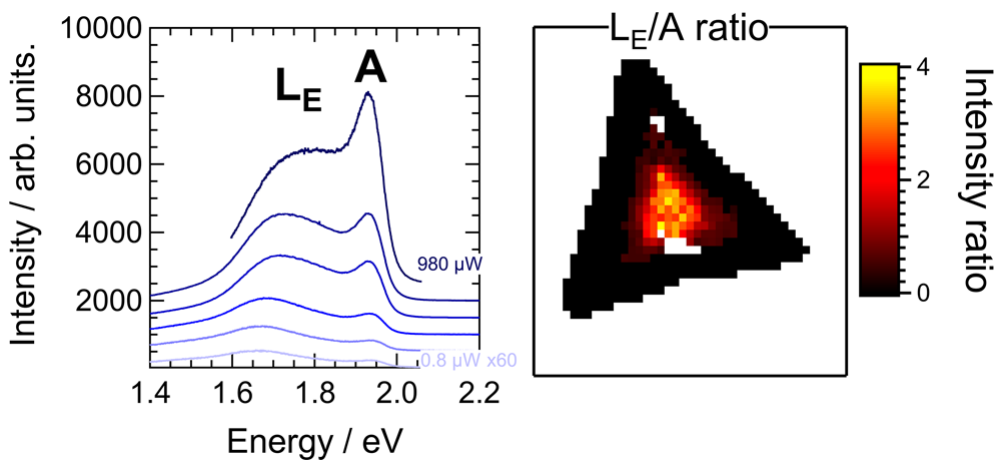

Alkali-metal-based synthesis of transition metal dichalcogenide (TMD) monolayers is an established strategy for both ultralarge lateral growth and promoting the metastable 1T phase. However, whether this can also lead to modified optical properties is underexplored, with reported photoluminescence (PL) spectra from semiconducting systems showing little difference from more traditional syntheses. Here, we show that the growth of WS_2_ monolayers from a potassium-salt precursor can lead to a pronounced low-energy emission in the PL spectrum. This is seen 200–300 meV below the A exciton and can dominate the signal at room temperature. The emission is spatially heterogeneous, and its presence is attributed to defects in the layer due to sublinear intensity power dependence, a noticeable aging effect, and insensitivity to washing in water and acetone. Interestingly, statistical analysis links the band to an increase in the width of the A_1g_ Raman band. The emission can be controlled by altering when hydrogen is introduced into the growth process. This work demonstrates intrinsic and intense defect-related emission at room temperature and establishes further opportunities for tuning TMD properties through alkali-metal precursors.

## Introduction

Two-dimensional transition metal dichalcogenides (TMDs) are of intense technological interest.^1,2^ Many, such as WS_2_ or MoS_2_, are typically found in the semiconducting 2H phase, with an indirect-to-direct band gap transition at the monolayer limit.^3,4^ This yields intense room-temperature photoluminescence (PL)^5^ in the visible range for use in next-generation optoelectronic devices. Exceptionally strong binding energies (around 0.3 eV for monolayer WS_2_ on SiO_2_^6^) mean that this room-temperature optoelectronic behavior is dominated by excitons,^7^ as well as other quasiparticles such as trions and biexcitons. Moreover, further applications are found in energy storage and catalysis, such as anodes in batteries^8^ or in the hydrogen evolution reaction,^9^ often via intrinsic or postsynthetic conversion to the so-called 1T phase, which has (semi-) metallic character in most cases.^10^

Considering this, substantial efforts have been placed on synthesizing large-scale TMDs with controllable phase, layer number, and properties. Synthesis by chemical vapor deposition (CVD) in particular offers the potential for scalable growth. The classical CVD synthesis involves the reduction and vaporization of transition metal oxides and a gas-phase reaction with sulfur or selenide (known as the vapor solid (VS) mechanism). But the different volatility of the reactants makes controlling the reaction difficult and remains a key technological barrier.^11^

Intense focus has therefore recently been placed on the use of alkali metals to enhance or tailor the synthesis of TMDs. While certain mechanistic details are still under debate,^12,13^ it is thought that the formation of monolayers can take place at the liquid–solid interface, following the melting of alkali-metal-salt-based precursors (the so-called vapor–liquid–solid (VLS) mechanism).^14,15^ This can lead to the growth of ultralarge layers. Moreover, potassium and lithium can drive the formation of the thermodynamically unfavored 1T phase in a manner intrinsic to the synthesis.^16,17^ For example, by using the alkali-metal salt precursors K_2_WS_4_ or K_2_MoS_4_, in the presence of H_2_, Liu et al. succeeded in growing 1T MoS_2_ and WS_2_ monolayers in spite of the large formation energy barriers.^16^ In the absence of H_2_, the 2H phase is formed.

In both phases, many properties are further determined by the presence and distribution of defects and inhomogeneities within the layer.^18−20^ One example is the trapping of excitons.^21−24^ Such localized excitons are of increasing research focus as potential sites for single-photon emission,^25,26^ as well as in quantum information,^27^ and valleytronics.^28,29^ Interestingly, while experiments have often required cryogenic temperatures for detection—although their emission from WS_2_ bilayers was seen up to around 180 K^30^—this has been extended to room temperature through postsynthetic patterning^31^ or substrate-induced strain and plasmonic enhancement.^32^

Beyond this, heterogeneities also provide a further framework for modifying or tuning other phenomena. Defects are known to lower the photoluminescent quantum yield and carrier mobility in pristine devices.^5,33^ On the other hand, by providing additional relaxation channels, defects can inhibit exciton–exciton annihilation and enhance photoluminescence intensity under high excitation powers.^34,35^ From an energy conversion perspective, defects also provide opportunities for both phase selectivity^36^ and enhancing the catalytic properties.^37^ Thus, understanding and tailoring defects in the monolayer, and how this can be controlled during or following synthesis, is of key research importance.

However, considering the growth in alkali-metal precursors for large-scale and phase-tunable layers, their role in the generation of defects and subsequent alteration of emissive properties is not well understood. Most work has shown that the photoluminescence does not differ significantly from more traditional CVD-grown layers.^38,39^ On the other hand, it has been shown that including NaOH in the synthesis can lead to p-doping of MoS_2_, overriding the intrinsic n-doping. Moreover, the solution processibility allows for additional dopants to be introduced.^40^ Nb-doped WS_2_ in this way led to the formation of localized states and a characteristic low-energy emission detectable at low temperatures.^41^

Herein, we show that the synthesis of WS_2_ monolayers starting from a potassium-salt precursor, K_2_WO_4_, can also alter the emissive properties at room temperature. This is seen through a pronounced low-energy emission without any postsynthetic treatment or plasmonic enhancement. The emission, denoted L_E_, is typically seen at around 1.7 eV and can dominate the characteristic emission from free excitons, A, located around 1.95 eV. The L_E_ band can be controlled by altering the temperature at which hydrogen is introduced into the growth. The emission is further found to be spatially heterogeneous and decays toward the flake edges. We attribute the emission to defects in the layer—as judged by a sublinear power dependence, noticeable aging effect, and insensitivity to mild washing of the sample in water and acetone—and propose further research to investigate its origin. Overall, this work establishes the use of alkali metals to tune the properties of TMDs beyond the 2H/1T transition for use in novel optical applications.

## Results and Discussion

We used WS_2_ as a model system. Considering the range of typical TMDs, WS_2_ is of particular interest since it has the largest band gap and a strong PL response at room temperature.^42^ This usually consists of a neutral exciton (the so-called A exciton) at around 2 eV and a charged trion (A^–^) at slightly lower energy.^43^ Due to spin–orbit splitting of the valence band, a second exciton, B, is found at around 2.4 eV, although its PL emission is normally of much lower intensity.^44^ These excitons are themselves significantly affected by the local environment. For example, CVD synthesis on Si/SiO_2_ substrates often leads to a red shift of the A exciton due to the presence of strain.^45^

**Figure 1:**
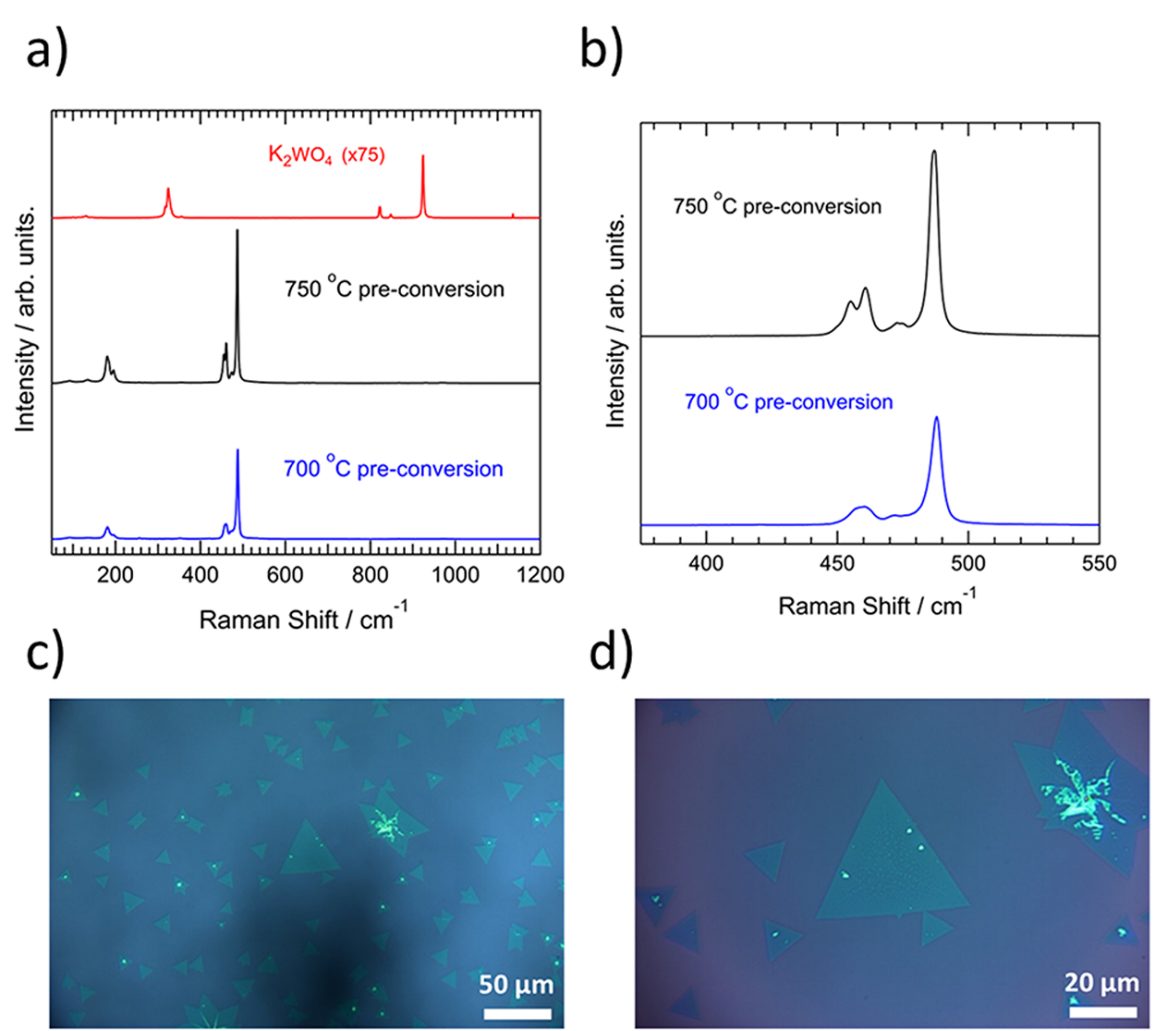
(a) Raman spectra recorded from powders of the initial precursor K_2_WO_4_ (red) and following preconversion at 750 °C (black) and 700 °C (blue), respectively. (b) Zoom of spectra for the preconversion at 750 and 700 °C from (a). (c, d) Optical images of the WS_2_ sample surface at two different magnifications following subsequent CVD reaction at 850 °C for 5 min.

WS_2_ was synthesized by CVD starting from a K_2_WO_4_ precursor using a two-stage process. First, K_2_WO_4_ powder was preconverted by reacting it with sulfur powder for 1 h under a N_2_/H_2_ atmosphere at 700 or 750 °C. The preconversion resulted in a noticeable change of color, from white to red-yellow. Figure 1a shows Raman spectra from the initial precursor and following preconversion at 700 and 750 °C, respectively. The initial precursor, K_2_WO_4_, was found to have prominent bands at 317 and 325 cm^–1^, as well as at around 822, 849, and 924 cm^–1^, respectively. These values are similar to those reported previously.^46^

Following preconversion, pronounced changes were observed in the spectra. As shown in Figure 1a, each of these characteristic bands of K_2_WO_4_ are no longer present. We refer to the work of Cordova et al.^47^ to analyze the changes in more detail. Therein, a Raman spectrum of bulk K_2_WS_4_ is reported, with prominent bands found in two regions at 180 and 200 cm^–1^ and 455, 459, 472, and, particularly, 487 cm^–1^. This band is assigned to a symmetric vibration of the WS_4_^–^ ions. The observed spectra under both preconversion conditions show strong similarities with this. In both cases, bands are found at around 181 and 196 cm^–1^, as well as four peaks closely matched to those in ref (47). In general, the positions are slightly upshifted in comparison to the reference (the bands for the 700 °C synthesis are found at 457, 461, 474, and 488 cm^–1^, respectively). Several other residual bands are found throughout the spectra of much lower intensity. This includes a band at 867 cm^–1^ that could indicate residual W–O bonds. However, its average intensity is only 0.2–0.3% of the peak at 488 cm^–1^. Overall, the Raman analysis suggests a significant conversion of K_2_WO_4_ to K_2_WS_4_ has taken place.

Figure 1b provides greater detail on the difference between the two preconversion conditions. This shows a zoom of the spectral region around 475 cm^–1^ for the reaction at both temperatures. It can be seen that at 750 °C this led to a spectrum exhibiting sharper peaks, with the two bands around 455 and 460 cm^–1^ clearly resolved from one another. Although this may suggest a more crystalline product, WS_2_ was also detected under this condition. Figure S1 shows different Raman spectra recorded under both conditions. The spectrum at 750 °C shows a noticeable reduction in intensity, with the E_2g_+2LA(M) and A_1g_ bands from 2H-WS_2_ clearly distinguishable at around 350 and 420 cm^–1^, respectively. A very weak band (with average intensity only around 0.3% of the 488 cm^–1^ band) is seen in all cases around 350 cm^–1^. The A_1g_ band at 420 cm^–1^ was then used as the determinant of WS_2_ in the measurements. Applying this, the detection of WS_2_ was found in approximately half of the spectra recorded following preconversion at 750 °C. The intensity of the 350 cm^–1^ band varied substantially in these spectra but averaged to 20% of the 488 cm^–1^ band. On the other hand, the A_1g_ WS_2_ band was not visible in any of the 700 °C spectra. Moreover, sharper peaks were also seen at times during heating at 700 °C (Figure S1). Consequently, the experiments proceeded with the product preconverted at 700 °C.

For the CVD reaction, the reacted precursor was thereafter deposited from aqueous solution onto a ±1 cm Si/SiO_2_ substrate by spin-coating. The substrate was first cleaned using argon plasma (70 W, 15 min) to increase the hydrophilicity. The reaction was carried out at 850 °C for 5 min in a N_2_/H_2_ atmosphere with a ratio of 1:10, and with H_2_ introduced from a temperature of 650 °C. Further details are included in the Methods Section. A schematic diagram of the synthesis setup is provided in the Supporting Information (Figure S2a). Figure 1c,d shows optical images from the Si/SiO_2_ substrate following the growth at two different magnifications. Isolated triangular-shaped layers can be seen on the substrate. The image in Figure 1c shows that a variety of different crystal sizes were obtained. In general, growth in the center of the substrate was characterized by a high density of small crystals, with the proportion of larger crystals (>10 μm) increasing further from the center. The largest crystals obtained during this growth were on the order of 50–100 μm (Figure S2b). Growth was also seen across the substrate. However, the size of the obtained flakes was not uniform. Additionally, while most flakes exhibited a triangular symmetry, this was not seen in all cases. Hence, the synthesis clearly also has further room for improvement.

We consider the reaction to proceed via the decomposition of K_2_WS_4_ to WS_2_. Liu et al.^16^ reported that this could lead to the growth of either the 1T or 2H phase depending on whether hydrogen is introduced into the growth chamber and reported reactions for the formation of 1T-MoS_2_. In both cases, K_2_S is formed as a byproduct, with the 1T product obtained via a potassium-containing intermediate, K_x_MoS_2_. A similar pathway is also expected for the formation of 1T-WS_2_.^17^

**Figure 2:**
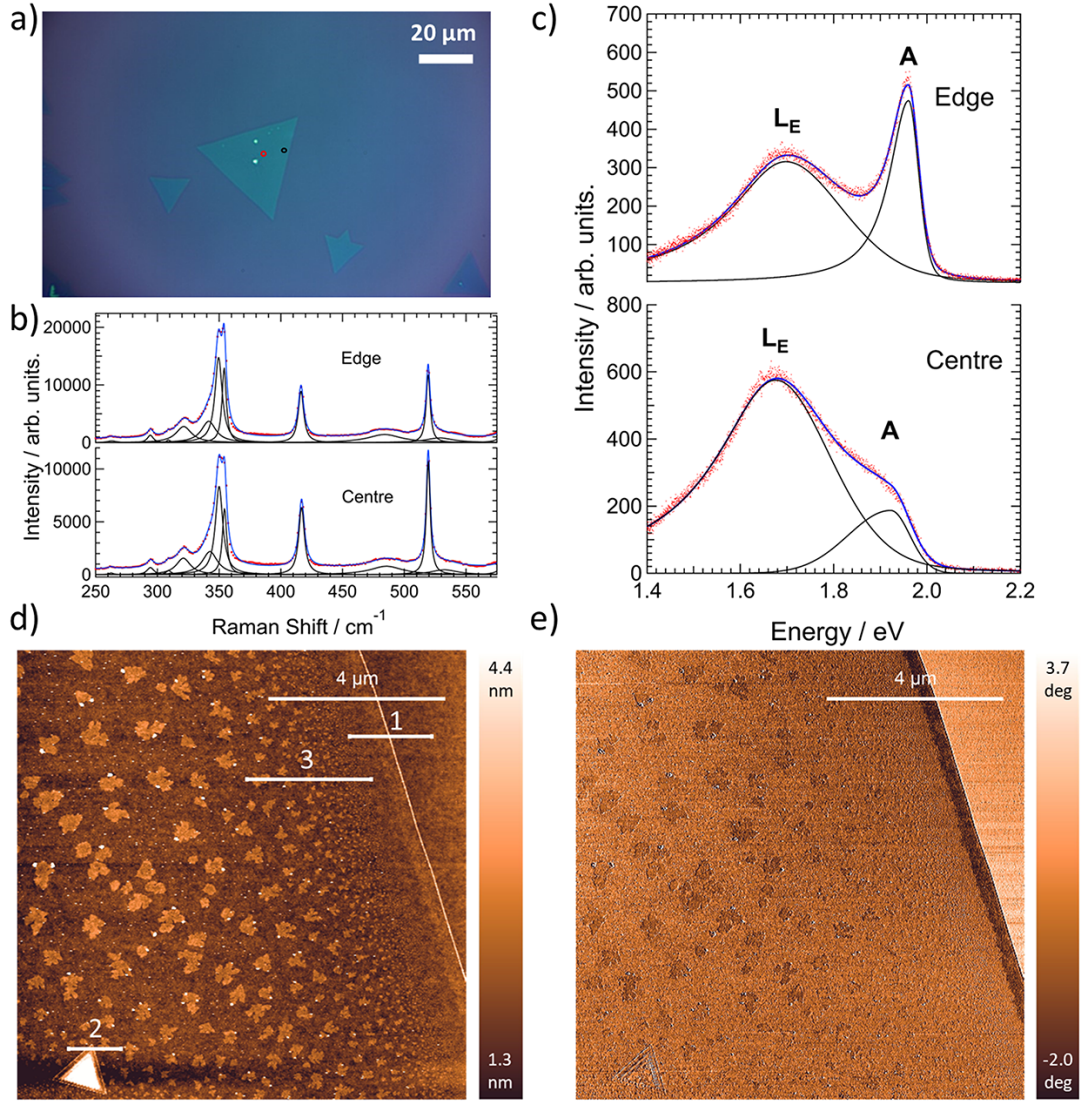
(a) Optical image from a synthesized flake, showing the positions of recorded spectra. (b, c) Raman (b) and PL (c) spectra and fitted curves from the positions marked in red (center) and black (edge) in (a), respectively. The raw data are shown in red with cumulative fitted curves and individual bands in blue and black, respectively. (d, e) AFM height (d) and phase (e) images recorded from the edge of a synthesized flake on the same sample.

Initial analysis of the synthesized layers was carried out with Raman and photoluminescence (PL) spectroscopy using 514 nm excitation (2.41 eV). Figure 2a shows an optical image of a characterized flake. In turn, Figure 2b shows Raman spectra recorded from its center and edge at the positions marked, respectively, by the red and black dots in Figure 2a. The blue curves in both cases indicate the results of multipeak fitting, with individual bands shown in black. The characteristic Raman bands of 2H-WS_2_ are clearly observed, namely, the A_1g_ band at around 417.5 cm^–1^ and the E_2g_ band around 355 cm^–1^ overlapped with the second order 2LA(M) band at around 350 cm^–1^.^47^ No indications of 1T-WS_2_ were found in the Raman spectra. The separation between the E_2g_ and A_1g_ bands was 62.4 cm^–1^ from the center of the flake, while the corresponding value from the edge was 62.2 cm^–1^. The average value across multiple flakes was also 62.2 cm^–1^. A value around 62 cm^–1^ is expected for monolayers^48,49^ and considering this, it was found that the A_1g_ band was located at 417.2 cm^–1^ on average, with the E_2g_ band at 355 cm^–1^, slightly lower than expected. As this band arises from in-plane vibrations, it is more sensitive to strain and its slight lowering may suggest tensile strain is present in the layer.

The separation between first-order bands suggests the crystals are monolayered. Further evidence for the number of layers is found through the intensity ratio between the 2LA(M) and A_1g_ bands,^48^ which is here equal to 1.3 and 1.7 from the center and edge, respectively (sample average of 1.4). This is slightly lower than found for a monolayer sample (though larger than the equivalent bilayer result) in ref (48) under 514 nm excitation. However, some dependency on the excitation wavelength was seen. Figure S13b shows a measurement on the same sample with a different setup (532 nm excitation), in which the 2LA(M)/A_1g_ ratio is greater than 3. Hence, it is therefore inferred that the observed crystals are single layer in general. It is interesting to note that the highest 2LA(M)/A_1g_ intensity ratio is seen under 532 nm excitation. Given that the 2LA(M) band is excited during a double-resonance process, this may imply that the 532 nm excitation (2.33 eV) is closer to the resonance condition of the B exciton here.^50^

However, concomitant measurements of the photoluminescence yielded unusual results. Figure 2c shows PL spectra recorded from the same positions at the center and edge of the flake. In both cases, the typical emission at around 1.95 eV is accompanied by a broad peak just below 1.7 eV. Moreover, the relative intensity of the low-energy PL band is greater in the center of the flake than at the edge. Accounting for the significant heterogeneity across the sample outlined below, it was found that the collective spectra could be fit most accurately by two split-pseudo-Voigt bands, with one representing the asymmetric emission around the A exciton (with likely contributions also from the free trion, A^–^), and the other the peak around 1.7 eV. Individual bands and fitted curves are again shown in black and blue in Figure 2c. The ratio of integrated intensities between these two bands was used to characterize the strength of the low-energy emission, yielding values of 2.2 and 6.7 for the edge and center, respectively. Further differences between the center and edge can be seen in the band positions, with the location of the emission from the A exciton being red-shifted in the center compared to the edge (by around 48 meV in this case).

It is known that bi- and tri-layered WS_2_ can exhibit emission in the same spectral region as observed here, through an indirect transition.^51^ However, given that the Raman analysis supports a single-layer assignment, it was considered that this is unlikely. We therefore tentatively assign it as a defect-related emission (L_E_) moving forward. A widely reported possibility is that the emission results from localized excitons captured by defects, although the strength of the signal at room temperature is unusual in the present case.

To further understand the origin of the low-energy band, the PL and Raman spectra were compared from multiple positions across the sample. This included flakes of a variety of shapes and sizes and optical images of each are included in Figure S3. Table S1 lists a series of Raman parameters extracted from each spectrum, along with the associated relative integrated intensity of the L_E_ band. One recent attempt to explain the origin of a low-energy PL band (assigned to localized excitons) in WS_2_ focused on two emergent Raman lines at around 410 and 385 cm^–1^ linked to sulfur vacancies and oxidation, respectively.^52^ However, neither band is detected within the present Raman spectra. Additionally, no link is seen between the relative intensity of the first-order LA(M) band, found at around 175 cm^–1^, and L_E_. It was previously suggested that this correlates more strongly with the position of the L_E_ band,^49^ but no relation is again found in the present data. As stated, shifts in the position of the first-order Raman bands can be representative of changes in strain or doping within the layer, but the relative intensity of L_E_ was not associated with the position of the E_2g_ band (sensitive to strain) or the A_1g_ position (senstive to doping).

It was seen that only the full width at half-maximum (FWHM) of the A_1g_ band showed a tentative link to the strength of emission, as displayed in Figure S4. This band originates from out-of-plane vibrations of the chalcogen atoms in the lattice, with a wider band in principle linked to reduced phonon lifetimes.^53^ It is additionally interesting to note that such a relationship is not seen with either the 2LA(M) or E_2g_ bandwidths. Here, the convolution of these two bands makes an accurate determination of widths challenging, but no strong link was seen with their sum.

It is of further interest that the L_E_ band does not seem to be strongly affected by the size or shape of the layer and does not seem to simply arise from flakes that are clearly of lower quality. The L_E_ band was seen from all of the flakes in Figure S3 apart from panel f, which has an unusual shape and large areas of multilayer growth. Additionally, the average values from the edge of the triangular flakes in panels a, c, e, and h (and Figure 2a) are essentially the same as those from the smaller triangular flakes (panels d and i), with values of 2.4 and 2.3, respectively. However, one possible influence of flake size is in the signal strengths from the center of the flakes. The band ratio was notably higher for the larger flakes (3.5) than for smaller ones (2.2, taken from panels d and g).

Tapping-mode atomic force microscopy (AFM) was also performed. Figure 2d,e shows height and phase images recorded from the edge of a flake. Both the height and phase images clearly identify the edge of the layer. Extracted height profiles associated with the lines in Figure 2d are provided in the Supporting Information (Figure S5). Typically, information on layer number can be obtained from AFM height analysis. However, in the present case, a noticeable difference in height was not observed between the TMD and bare substrate (profile 1 in Figure S5). In contrast, measurements from the multilayer region at the bottom of Figure 2d did give an accurate height difference between two WS_2_ layers (yielding a value of around 0.9 nm) as seen in profile 2 of Figure S5. It has previously been demonstrated that differences in hydrophobicity between TMD layers and bare SiO_2_ substrates can preclude accurate determination of layer heights, due to capillary forces acting between the tip and sample.^54^ In the same study, it was also shown that the image contrast and size of the projected step height depended on the scanning parameters used, with contrast inversion also seen under certain conditions. Indeed, in our case, successive measurements of a different flake produced height contrasts of opposite sign (Figure S6). Analysis from a separate sample (that used in Figures 4–6) also produced anomalous results, with obtained heights of around 0.4–0.6 nm (Figure S7), still lower than expected. The correct height variation between two TMD layers was again seen.

**Figure 3:**
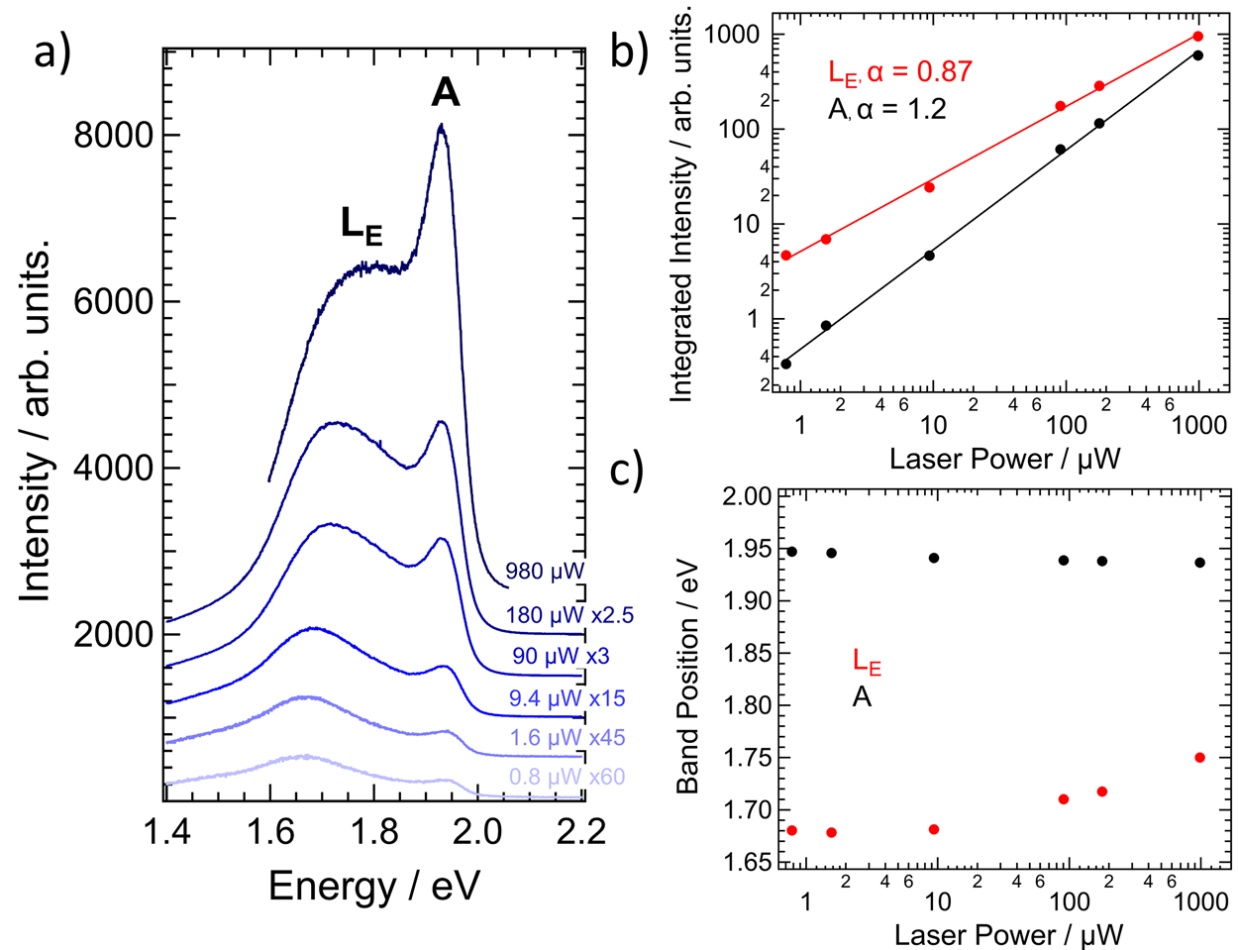
(a) Power-dependent spectra recorded from a WS_2_ monolayer. (b) Graph of the extracted integrated intensity of the A and L_E_ bands against excitation power, respectively, along with the fits to the linearized data. The displayed exponents, α, are averaged from three measurements on different flakes. (c) Corresponding graph of the L_E_ and A band positions against excitation power.

**Figure 4:**
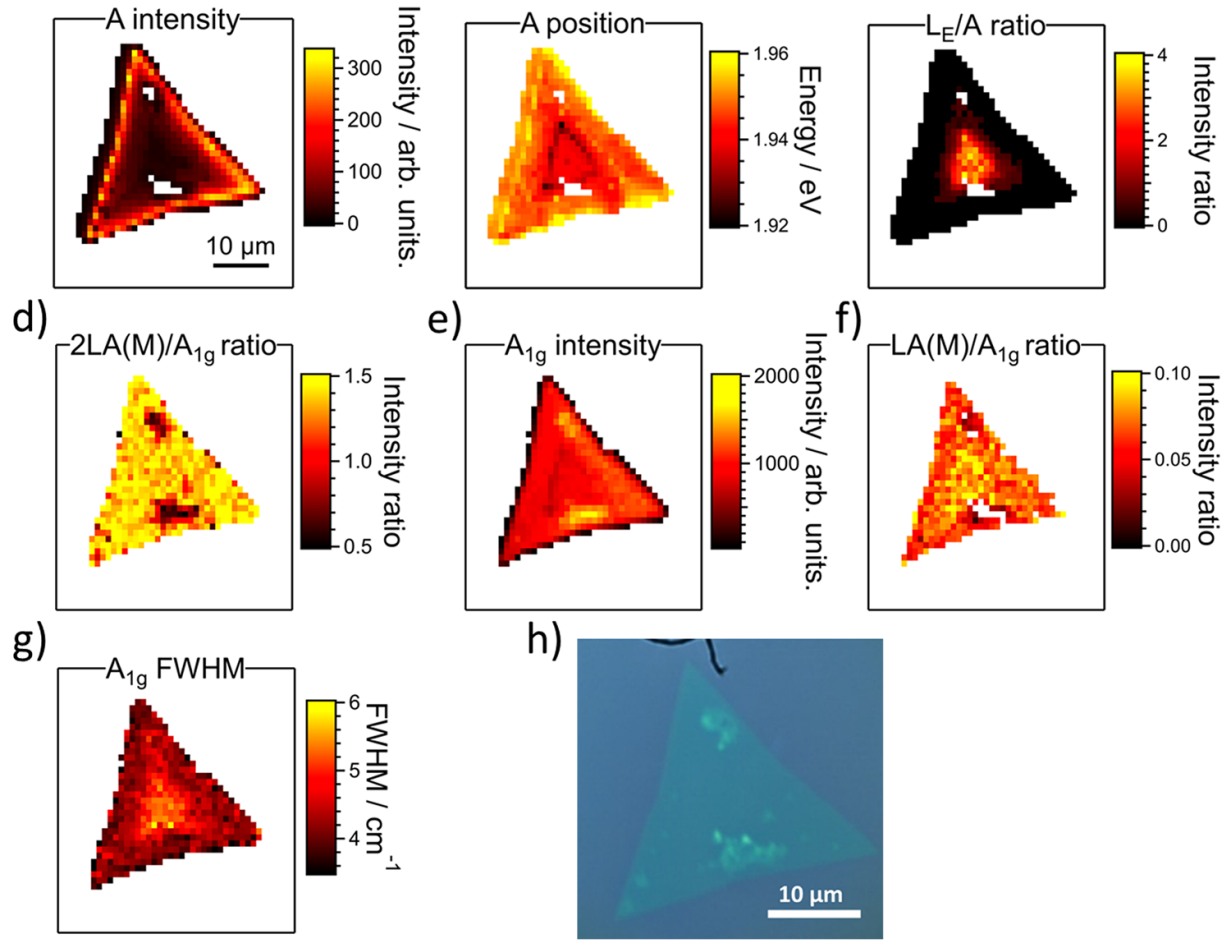
(a–c) PL maps of the integrated intensity of the A exciton, band position of the A exciton, and integrated intensity ratio of the L_E_ and A bands, respectively. (d–g) Raman maps of the 2LA(M)/A_1g_ intensity ratio, A_1g_ intensity, LA(M)/A_1g_ intensity ratio, and FWHM of the A_1g_ band, respectively. (h) optical image of the mapped flake.

**Figure 5:**
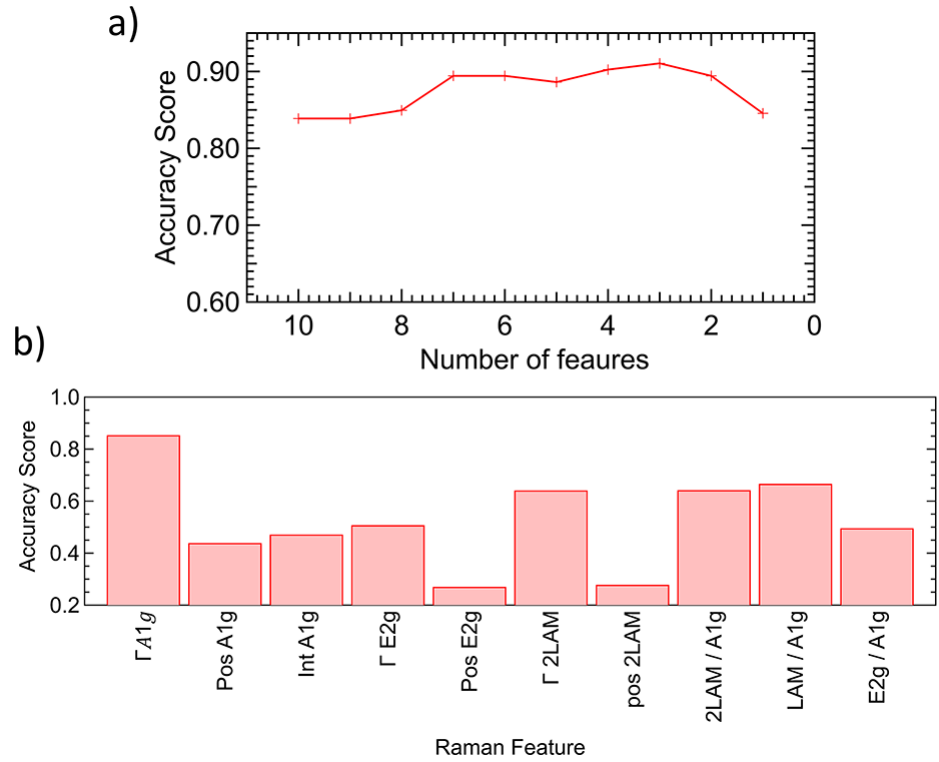
(a) Graph showing the test accuracy score against number of features for a logistic regression model to predict the presence of the L_E_ band using extracted Raman parameters as features. (b) Test accuracy score for the model when trained on a single Raman feature.

**Figure 6:**
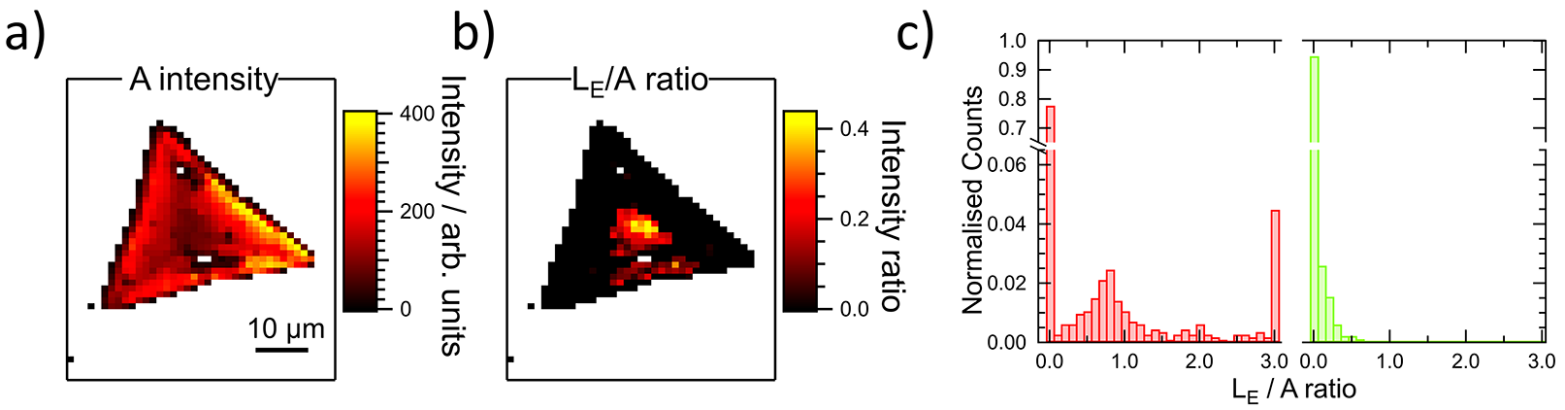
(a, b) Maps of the A exciton intensity and ratio of the L_E_ and A bands integrated intensity, respectively, following aging in air for around 2 months. (c) Histograms of the relative integrated intensity from before (red) and after (green) aging in air.

However, the topographic analysis yields further information. Figure 2d shows the presence of additional material on top of the crystal, the lateral size of which appears to grow moving away from the crystal edge. Line profile 3 (Figure S5) illustrates that the height of such material is around 0.6 nm. However, it is noted that the phase image in Figure 2e shows differences between this and WS_2_. The lower phase value of this material indicates a different environment for the tip and distinguishes the material from WS_2_. Chemical identification is not possible through AFM, but additional material has also been seen in previous AFM images from TMDs grown through a VLS mechanism.^38^ It is also notable that its distribution shows some similarities to the distribution of the L_E_ band. The presence of organic adlayers was proposed in ref (31) as the mechanism for a similar low-energy emission through a charge-transfer exciton. Yet unlike that study, no e-beam irradiation was performed in the present case. One possibility is it could arise from a reaction byproduct, such as K_2_S. But contamination was also not always detected on other samples, such as that used in Figures 4–6, synthesized under the same conditions (Figure S7). Hence, we find any influence on the optoelectronic properties of the crystals inconclusive at this stage.

The nature of the L_E_ emission was further probed through laser power dependence measurements. Figure 3a displays spectra recorded at the same position with excitation powers ranging over approximately 3 orders of magnitude. This shows that the relative intensity of the L_E_ band decreases as the power is increased. At lower excitation intensities, the L_E_ emission is by far the brightest in the spectrum. But it is increasingly superseded by the A exciton emission at higher powers (the integrated intensity ratio between the two bands drops from 14 to 1.6 in this example.) Figure 3b shows the extracted integrated intensity for both bands against excitation power on a log-log scale, along with fits to the linearized data. The average value for the exponent, α, was 0.87 and 1.2 for the L_E_ and A bands, respectively. In theory, the defect-induced L_E_ emission should be sublinear,^55,56^ due to saturation of defects in the layer. The value is also clearly below 1 here and significantly less than for the A exciton. This provides good evidence supporting the assignment of the low-energy peak. Since a complete saturation of the L_E_ band is not seen, it may also suggest a high density of defect sites in the sample, or that it arises from a free-to-bound or donor–acceptor pair recombination.^57^

Figure 3c also shows the change in the position of the bands with excitation power. While the A exciton shows a small decrease in position at low powers and is then broadly static, L_E_ shows a blue shift, increasing by about 30–40 meV per decade of laser power. The positions at lower powers may suffer from greater uncertainty in the fitting. Similar behavior was seen at low temperatures from localized excitons in WS_2_ monolayers in our previous work,^55^ as well as by others.^31^

Correlated PL and Raman hyperspectral mapping were then recorded. Note that this was performed on a second sample, synthesized using the same procedure and yielding qualitatively comparable results to the one discussed above (see Figure S8b). Two maps were recorded on the sample at a resolution of 1 × 1 μm^2^. Figure 4a displays a map of the A exciton integrated intensity from a single flake, for which an optical image is given in Figure 4h. There is significant variation across the flake, with much stronger emission from the A exciton toward the edges than in the center. This itself is a common phenomenon in TMD monolayers synthesized by CVD.^42^ In the center of the sample, the exciton intensity drops to around 10% of the value close to the edges. Again, this variation is of a similar magnitude to what has been reported previously.^42^ A difference is also seen in the position of the A exciton, as is shown in Figure 4b. There is a subtle blue shift of 10–20 meV moving from the center to the edge of the flake, with typical values of around 1.94 and 1.95 meV, respectively.

Figure 4c shows the integrated intensity ratio between the L_E_ and A exciton bands across the flake. In agreement with the spectra in Figure 2c, the values are greatest in the center. Here, the integrated intensity of the L_E_ band is around 3 times greater than the A exciton. Moving outward, the L_E_ band decreases in intensity and then appears as an asymmetric tail to the A exciton. In contrast to Figure 2, no emission was detected around the edges of the flake on this sample. Moreover, the boundary between the two regions is also distinguished by the lowering of the A exciton position as seen in Figure 4b. In order to avoid spurious fitting to the background, a threshold filter was applied to only include the L_E_ band in the fitting procedure if the estimated value was greater than 5% of the initial estimated values for the exciton peak. Thus, the band may in fact be present beyond the limit included here. In cases close to this limit, establishing the origins of any low-energy asymmetry in the PL spectrum is challenging.

Broadly similar features are seen from a second position, shown in Figure S9. Here, the lowering of the A exciton position extends over a wider area around the center of the flake. In both cases, the PL outside this region is characterized by further strengthening of the A exciton emission intensity, and an increase in the PL position, alongside the absence of the L_E_ band. These changes are characteristic of areas of higher optical quality, and so may define the edge of the defective region.

Further efforts were then made to correlate the emission with characteristics extracted from the Raman measurements. Figure 4d–f shows maps of the 2LA(M)/A_1g_ ratio, the absolute A_1g_ intensity, and the intensity ratio of the LA(M) and A_1g_ bands, respectively. It is seen that the intensity ratio between the 2LA(M) and A_1g_ bands is consistent between the center and edge of the flakes. We therefore consider that the flake is monolayered, apart from two clearly multilayer regions (which also did not give a PL signal). Further, Raman maps are given in Figure S8c–e for the sum of the E_2g_ and 2LA(M) FWHM, A_1g_ and E_2g_ separation, and position of the A_1g_ bands, respectively. There is also no change in the separation between the two first-order bands (Figure S8d). However, there may be a tentative link to the absolute Raman scattering intensity. As shown in Figure 4e, the intensity of the A_1g_ band does change across the flake. In general, the overall Raman scattering intensity shows a similarity in distribution to the position of the A exciton. Individual spectra are extracted from 4 points in the map, shown in Figure S10a. As illustrated by the dark blue curves in Figure S10b,c, around the transition between the regions with and without a strong L_E_ band, there is a notable drop in both the position of the A exciton (Figure S10b) and the intensity of the A_1g_ band (Figure S10c). The green curves in Figure S10b,c are from the center of the flake, with both parameters then recovering to their values at the edge positions (shown by the light blue and black curves in Figure S10b,c). These changes are more evident in Figure S9b,e, in which the region of lower A exciton position and A_1g_ intensity is greater than the example in the main text.

Next, Figure 4f shows the intensity ratio of the LA(M) and A_1g_ bands. In accordance with the point spectra, this also does not appear to correlate with the L_E_ band. Finally, Figure 4g shows the distribution of the A_1g_ FWHM across the flake. The map suggests the tentative link between L_E_ intensity and the FWHM A_1g_ is also visible, with the peak broader within the central region. This variation was again not seen in the 2LA(M) or E_2g_ bands (Figure S8c). Such an effect is not large and amounts to a difference of approximately 1 cm^–1^ between the center and edge regions. Nevertheless, the links between the Raman and PL data suggest there may be structural changes arising from the growth and warrant further investigation.

We then attempt to correlate the spectral changes seen in the maps in more detail. The width of the A_1g_ band was plotted against the L_E_/A intensity ratio from both maps (Figure S11a). Only a weak correlation was observed. A stronger link was seen, however, between the intensity of the A exciton and the relative magnitude of the L_E_, as is shown in Figure S11b. L_E_ emission could only be detected in positions where the exciton was less than around 20% of its maximum value. The distribution between the A exciton position and L_E_ relative intensity is shown in Figure S11c. As suggested in Figures 4b and S9b, a lowering of the exciton position is first linked to a modest increase in the L_E_/A ratio. However, a change is observed around an L_E_/A value of 1–2, after which the A exciton position recovers to close to its initial value. This suggests that two different regimes may be present and this is further implied by the A_1g_ intensity. Figure S11d shows the distribution of the A_1g_ intensity against the A exciton position. For the bulk of the data, an increase in A_1g_ intensity is associated with a very slight lowering of the A exciton band position. However, a clustering of further points is found below this, associated with the center of each flake. Overall, the correlations highlight the links between the Raman and PL data and may suggest a structural origin for the L_E_ band.

To further investigate any links between the Raman structural data and the presence of the L_E_ band, we next used the map data to predict the presence of the L_E_ band through multifeature logistic regression. Here, the goal was only to predict the presence of the L_E_ band above a certain threshold value, not its relative integrated intensity. First, the data was split into training and testing sets and a total of 10 Raman features were selected to include in the model. This included the relative intensity of LA(M), E_2g_, and 2LA(M) bands (normalized against A_1g_) and the positions and widths of the A_1g_, E_2g_, and 2LA(M) bands. The absolute intensity of the A_1g_ band was also included. The model was then trained on the training data. The performance was determined using the ratio of correct predictions to total predictions (accuracy score) from the test data, which yielded a value of 0.84 when all features were included.

Next, a recursive feature elimination was applied to determine the most important parameter. Here, the least significant Raman parameter was iteratively removed from the model before being retrained using the remaining features. Figure 5a shows the accuracy score generated by the model as the number of features is reduced. Ultimately, this confirmed that the width of the A_1g_ band was the most important parameter. Moreover, the accuracy of the model was 0.85 when this single feature was included, although it peaks at a value of 0.91 (when the A_1g_ width, E_2g_ position, and E_2g_/A_1g_ ratio are used).

We further performed logistic regressions using each of the Raman parameters individually, with the results shown in Figure 5b. This shows the accuracy score of the model in predicting the presence of the L_E_ band in each case. Given that the classification is binary, only the width of the A_1g_ band gave a significant result. Both the E_2g_ position and E_2g_/A_1g_ intensity ratio scored less than 0.5 individually, suggesting their previous inclusion in the mutlifeature model was due to overfitting.

Thus, the analysis confirms two things. First, the width of the A_1g_ band is the single most important Raman parameter in terms of understanding the L_E_ band. Second, it demonstrates a link between the Raman and PL data sets and may imply the primary limitation is in the sensitivity of the Raman measurements. It also confirms that the L_E_ band does not arise through doping or strain, at least to the extent that can be determined here.

The low sensitivity is itself not unexpected as previous work has also shown that large changes in PL emission did not cause large variations in the Raman response.^58^ However, recent work has also shown that the PL and Raman characteristics of MoS_2_ can be linked and understood through more complex machine learning methods than used here.^59^ The distributions in Figure S11c,d also suggest other more complex links between the data sets may be present. More detailed analysis could be required to further link the Raman and PL data and should be a topic for subsequent research.

Maps recorded on the same sample approximately 2 months after synthesis, following storage in air, indicated a pronounced aging effect. Figure 6a shows a map of the A exciton intensity on the same flake as Figure 4a. It is clear that the intensity distribution is qualitatively similar; greater intensity is found around the flake edges and with a similar magnitude to Figure 4a. However, it is notable that the A exciton emission is relatively strengthened in the center, where the integrated intensity is only around 3–4× weaker than at the edges. Figure 6b reproduces the map of the L_E_/A integrated intensity ratio. Here, the region in which no signal can be detected has expanded, with the band now only located within the central region. Furthermore, the integrated intensity relative to the A exciton has also decreased, with the greatest values within this central region of around 0.5. Histograms of the L_E_/A integrated intensity ratios from the fresh and aged samples (considering two maps in each case) are shown in Figure 6c in red (fresh flake) and green (aged flake), respectively. For clarity, values greater than 3 have been binned together. It can be seen that around 78% of pixels did not have a detectable L_E_ band in the maps taken directly after synthesis (note that pixels where PL was not detected are not included). This value increases to around 95% in the aged sample. Moreover, among the spectra where L_E_ was detected, the distribution initially exhibited a peak in the relative intensity around 0.7, with almost all bins populated above this and around 4% of spectra having values equal to or greater than 3. In contrast, the aged sample exhibits no peak and shows a continuous decrease in bin populations. No integrated intensity ratios greater than 0.6 were recorded on the aged sample.

An aging effect further suggests that the L_E_ emission does not arise through any indirect emission from multilayers. It is more likely that the native defects in the sample that contribute to L_E_ have undergone a chemical transformation, such as oxidation. Additionally, to consider whether the L_E_ band could arise from contaminants on the sample, mild and careful washing in acetone and milli-q water was performed. This led to a clear redistribution of material on the substrate. PL spectra recorded from two flakes before and after washing are shown in Figure S14. However, only limited changes are seen to the relative intensity of the L_E_ band, although a notable blue shift was seen in its position.

**Figure 7:**
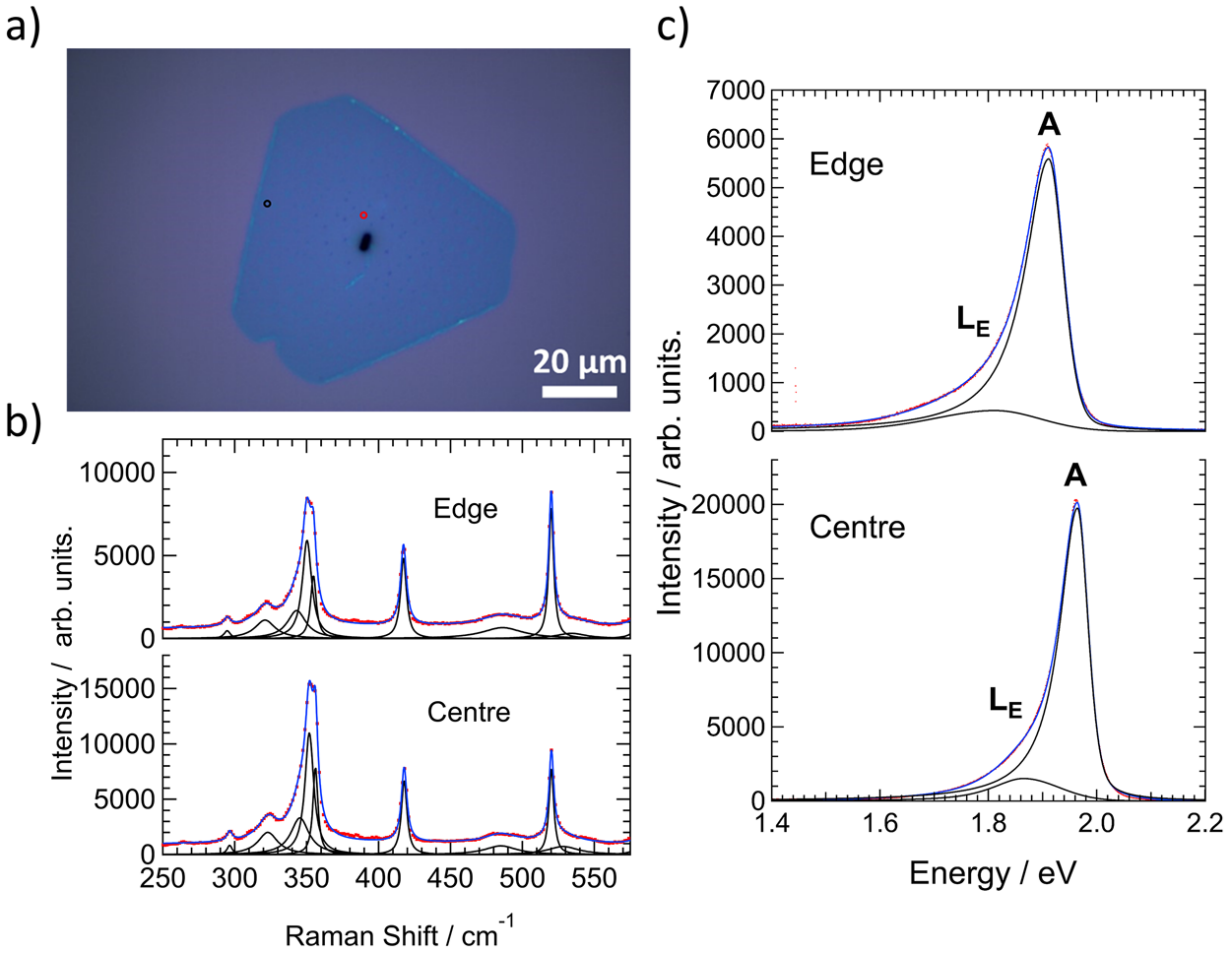
(a) Optical image of a flake synthesized with hydrogen introduced at the growth temperature (850 °C). (b, c) Raman and PL spectra recorded from the positions marked in (a) representing the center (red) and edge (black) of the flake, respectively. In all cases, the spectra are shown in red alongside fitted curves in blue, with individual bands shown in black.

Finally, we show that the presence of the L_E_ band can be controlled by changing the point at which hydrogen is introduced. If H_2_ is introduced once the growth temperature is reached (850 °C), instead of during the temperature ramp (650 °C), then the L_E_ band is significantly suppressed. Figure 7a shows an optical image of a flake synthesized under this condition. Here, the growth time was increased to 10 min as no growth was visible after 5 min. All other conditions were left unchanged. Two points are highlighted by the red and black circles, from which Raman and PL measurements were recorded. Figure 7b displays Raman spectra in red, cumulative fitted curves in blue, and individual bands in black from the center (red dot) and edge (black dot), respectively. The characteristic 2H-WS_2_ bands are seen from both positions. The corresponding PL spectra are displayed in Figure 7c. The absolute PL intensity is greatly enhanced compared to the respective image in Figure 2c. This was a result seen in general when comparing multiple flakes across the two samples. In further contrast to the synthesis when H_2_ is introduced earlier, the intensity within the center of the flake is now higher than at the edges. The intensity of the A exciton was on average around 3 times higher in the center than at the edge, when averaged across all measurements. Moreover, in both positions the L_E_ band is now significantly suppressed and shifted to higher energy such that it is difficult to separate from any contribution from the trion, A^–^. Hereby, we assign the emission to the L_E_ band and consider the associated values to be a maximum of the possible contribution. The average value of the integrated intensity ratio between the L_E_ and A bands was 0.15 (center average = 0.13, edge average = 0.17) when H_2_ was introduced at 850 °C, while it was 2.4 (center average = 2.7; edge average = 2.0) when the H_2_ was introduced at 650 °C. The position of the L_E_ and A bands were respectively 1.83 eV (center = 1.86 eV; edge = 1.78 eV) and 1.94 eV (center = 1.96 eV, edge = 1.91 eV). The equivalent values when H_2_ was introduced at 650 °C were 1.69 eV (center = 1.70 eV; edge = 1.69 eV) and 1.94 eV (center = 1.93 eV, edge = 1. 95 eV). It can therefore be seen that the position of the A exciton is broadly the same between the two synthesis conditions. Given that the heating and cooling rates were the same, this may suggest the lower-than-expected position of the A exciton is a result of strain.

### Discussion

Alongside the sublinear power dependence, the aging effect and insensitivity to mild washing point to a defect-related mechanism for the L_E_ band emission. It is known that defects can be introduced extrinsically or be intrinsic to the synthesis. One example of extrinsic modifications to the optical properties is through focused ion-beam milling.^60^ Therein, it was shown that this introduced spatially varying changes and a low-energy emission associated with defect-localized excitons at around 1.8 eV visible at room temperature. The emission was assigned to sulfur vacancies in the lattice and other work has also pointed to chalcogen vacancies as a source of localized excitonic emission.^21,61^ Oxygen-passivated interstitial defects have also been considered as the source of single-photon emission via localized excitons in WSe_2_.^62^ In our case, it is interesting to note the changes in the spectra caused by delaying the H_2_ introduction. That this leads to distinct changes could suggest different reaction pathways at lower and higher temperatures, with the reaction only proceeding in a reductive atmosphere, and the lower temperature growth generating more defects. We have also found little to no growth on samples where H_2_ was not introduced under otherwise identical parameters.

Substitutional dopants in the layer can also alter the emissive properties. Co-doping of WS_2_ by Cr, Fe, Nb, and Mo was found to introduce impurity states. This again led to a low-energy room-temperature emission associated with localized excitons,^63^ with a peak at around 150 meV from the A exciton. Similar behavior assigned to localized excitons has also been seen following Nb substitutional doping of WS_2_,^64^ with emission centered around 1.4–1.6 eV, again at room temperature. In this sense, it is notable that the reaction proceeds via a potassium-containing precursor, in which previous reports have suggested proceeds through potassium-containing intermediates. Thus, one possibility is that potassium has become doped within the layer. But theoretical calculations have suggested that substitutional doping by group 1 elements is energetically unfavorable at either the tungsten or sulfur sites^65,66^ (although other work has suggested the opposite).^67^ We also find no consistent evidence for additional bands in the Raman spectra that could be consistent with this,^41,64^ with Raman also suggesting the flakes remain in the 2H phase. The separation of the L_E_ band from the A exciton (often 200–300 meV) and its strength at room temperature indicate that a deep potential would be required for the localization. Another possibility is that the emission arises from a free carrier transition, for instance between the conduction band and acceptor levels above the valence band. Distinguishing between these options would require further analysis, such as time-resolved techniques (e.g., transient absorption spectroscopy) or temperature-dependent photoluminescence and is a topic for further research beyond the main objective of this paper.^68−70^ Further work could explore in greater detail the role of hydrogen in directing the growth of alkali-metal-containing precursors, both in terms of the emissive properties and polymorph selectivity.

## Conclusions

The synthesis of WS_2_ from a potassium-based precursor is associated with unusual emission at room temperature, found around 200–300 meV below the A exciton. We hypothesize that the emission arises from a defect-related recombination, such as localized excitons bound to defects. In this case, unlike many other recent reports of room-temperature localized emission, its presence is intrinsic to the synthesis and also does not require plasmonic enhancement. A second possibility is the emission arises from a free-to-bound recombination, which could also have implications for previous studies. The band is found to be spatially heterogeneous, and decays when moving toward the flake edges. The emission can be suppressed by introducing hydrogen at the growth temperature (850 °C) rather than during the temperature ramp (650 °C). Attempts to link the band to structural changes through Raman analysis only suggest a link to the width of the A_1g_ band at around 417 cm^–1^. Efforts to tune the parameters of the emission through the synthesis conditions may open the possibility for tailored emission from monolayer WS_2_ from 2 eV until the near IR.

## Methods

### Synthesis

The two-stage synthesis involved a preconversion followed by subsequent WS_2_ growth. Both were conducted in a 3-zone split tube furnace (MTI corporation). For the preconversion, approximately 15 mg of K_2_WO_4_ (Alfa Aesar) was loaded into the third heating zone, with roughly 250 mg of sulfur powder loaded upstream into the first zone. The system was purged with N_2_ and then heated at 100 °C under vacuum for around an hour. Thereafter, a N_2_/H_2_ gas mixture was introduced at an approximate ratio of 1:10 and the first and third zones were gradually heated to 200 °C and 700/750 °C, respectively, over a period of 50 min. The temperature was thereafter held for 1 h before natural cooling to room temperature.

To synthesize the layers, silicon substrates with a 280 nm SiO_2_ layer (approximately ±1 cm^2^) were gently washed in Milli-Q water and dried under nitrogen, before being cleaned by argon plasma for 15 min at 70 W. Immediately thereafter, 10 μL of an aqueous solution containing the preconverted reactant (approximately 1 mg/mL) was deposited using spin-coating (2000 rpm for 1 min). This modified substrate was then loaded into the third zone of the chamber. Thereafter, the system was purged with N_2_ and heated at 100 °C under vacuum for about 1 h, before N_2_ was again introduced with a flow rate of about 132 mL/min. The first and third zones were then heated to 200 and 850 °C over 50 min. Unless otherwise stated, once a temperature of 650 °C was reached, the N_2_ flow rate was reduced to about 120 mL/min and H_2_ was introduced to yield an approximate H_2_/N_2_ gas ratio of 1:10. The temperature was held at 850 °C for 5 min (unless otherwise stated). After this, the H_2_ flow rate was reduced to zero and the N_2_ rate significantly increased. The chamber was opened to allow for rapid cooling to room temperature. In many cases, a second Si/SiO_2_ substrate (also cleaned by argon plasma) was positioned ±1 cm downstream from the first. However, typically limited or no growth was seen in this sample, so it is not discussed in more detail here.

Although no additional sulfur was added to the chamber for the growths included in this paper, it is also considered to still be necessary for the synthesis. Following the preconversion, noticeable deposits of sulfur were found within the chamber, typically at the beginning of zone 2 (see Figure S2a). Multiple experiments (10 or more) could then be performed reproducibly before the growth failed. Similarly, growth in a prior cleaned chamber (through heating the 1st and 2nd, and 3rd zones to 650 and 950 ^o^C for an hour under H_2_/N_2_ ratio of 1:10) also did not lead to the growth of a monolayer. Growth with 25 mg of S added to the 2nd zone led to similar growth with notable emission from the L_E_ band (Figure S12), although growth under these conditions is not discussed further here.

### Characterization

Most Raman and PL measurements were taken on an Invia Microscope (Renishaw) using a continuous-wave argon laser (514 nm) excitation and a 100× objective (NA = 0.75). Excitation powers varied from approximately 1 to 1000 μW. Raman and PL maps were taken at a resolution of 1 μm with 1 s acquisition at powers of around 200 and 10 μW, respectively. Separately, some measurements (Figures 1a,b and S13) were taken on a Horiba LabRam HR800 micro-Raman system with a continuous-wave Nd-YAG laser (532 nm excitation). All data was processed using IGOR Pro apart from the power-dependent measurements that were processed using Fityk. The reported exponents from the power-dependent measurements were averaged across three measurements on different flakes. Due to the reduced spectral range, the PL maps were fit with a combination of a Gaussian and split-pseudo-Voigt for the L_E_ and A bands, respectively. In other cases, the PL spectra were fit with two split-pseudo-Voigt curves. All Raman data was fit using Lorentzian curves. Band intensities extracted from the maps were normalized against the intensity of the Rayleigh emission.

For the maps, lateral misalignment between the PL and Raman measurements was accounted for by producing images of the absolute intensity of the A_1g_ mode and PL emission at around 1.97 eV. Cross sections in the *x* and y directions were taken to determine any misalignment. Both the Raman and PL data were housed in two-dimensional arrays, with each column representing a single spectrum. Columns of the raw Raman data were then shifted to account for the misalignment. That is, for a misalignment in the y direction, the data in the columns corresponding to row m in the Raman map were placed in those representing row *m* + *n*, where *n* was the degree of misalignment. Corresponding maps or rows around the edge of the map were set to zero if the required data was not recorded (i.e., it was beyond the edge of the map). The alignment was checked by again extracting profiles in the *x* and *y* direction and comparing them to the PL data.

A 3 × 3 Gaussian filter was applied to all maps before performing the logistic regressions. A threshold filter of 0.2 was then applied to the resultant L_E_/A integrated intensity ratio. Pixels that met this requirement were assigned a value of 1 with 0 given otherwise. First removing those corresponding to the bare substrate, 80% of the remaining pixels were randomly assigned as training data for the logistic regressions, with the remaining 20% used as testing data. The performance of each logistic regression was determined using an accuracy score that gives the ratio of correct predictions to total predictions. Quoted accuracy scores are taken from the testing data only.

AFM measurements were performed on a Multimode instrument (Bruker) with a Nanoscope V controller using tapping and peak-force tapping modes. Images were processed using Gwyddion.

## Supporting Information Available

Additional Raman spectra from the preconversion of K_2_WO_4_; details of the experimental setup and additional image from the WS_2_ substrate; extracted Raman parameters from point spectra; optical image from the flakes used in the point spectra analysis; graph of FWHM of A1g against the Le/A integrated intensity ratio from point spectra; extracted height profiles from Figure 2d; successive AFM images from a WS_2_ flake; AFM images from the same sample as Figures 4 - 6; additional Raman and PL maps from a WS_2_ flake; additional Raman and PL maps from a different flake; extracted PL and Raman spectra from the map in Figure 4; correlation plots from the map data; additional PL maps from a sample with added sulfur; Raman and PL spectra with 532 nm excitation; and PL spectra recorded before and after washing (PDF)

## Abbreviations

PL: photoluminescence
WS_2_: Tungsten disulfide
TMD: transition metal dichalcogenide
MoS_2_: molybdenum disulfide
FWHM: full width at half-maximum
K_2_S: potassium sulfide
