# Supplementary material for: Unusual Defect-Related Room-Temperature Emission from WS2 Monolayers Synthesized through a Potassium-Based Precursor
Source: ACS Omega. 2023 Oct 3;8(41):37958–70. doi: 10.1021/acsomega.3c03476 (PMC10586178; doi:10.1021/acsomega.3c03476)
Supplement: Supplementary file 1 — ao3c03476_si_001.pdf [file ao3c03476_si_001.pdf]

Unusual defect-related room temperature emission from WS2 monolayers synthesized through a potassium-based precursor. Supporting information.

Peter Walke, Reelika Kaupmees, Maarja Grossberg-Kuusk, and Jüri Krustok*

Department of Materials and Environmental Technology, Tallinn University of Technology, Ehitajate tee 5, 19086 Tallinn, Estonia

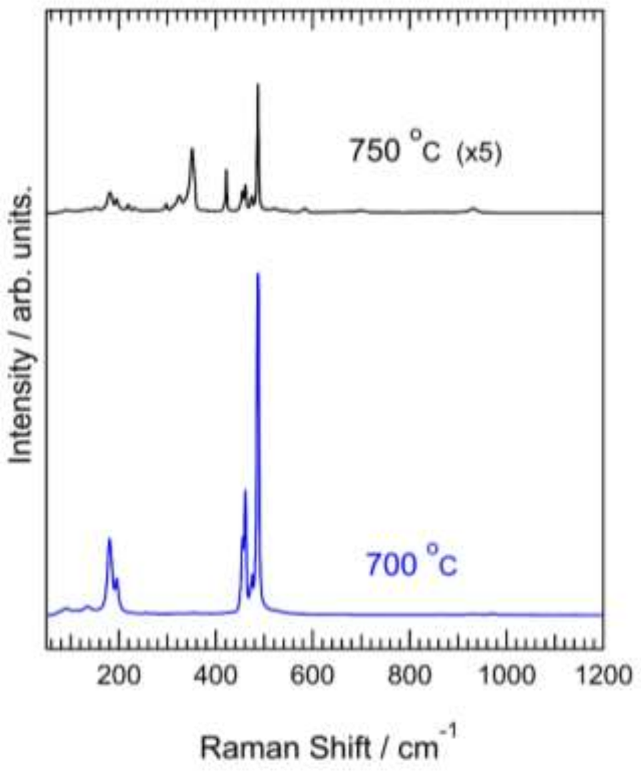

Figure S1. Additional Raman spectra from the pre-conversion of K2WO4 at 700 ºC (blue) and 750 ºC (black), respectively.

Figure S2 gives detail of the experimental setup. For the pre-conversion, the K_2WO_4 powder was placed in zone 3 with sulfur powder placed upstream in zone 1. For the subsequent growth, the deposited sample was again placed in zone 3. For the results presented in figure S12, additional sulfur powder was placed in zone 2.

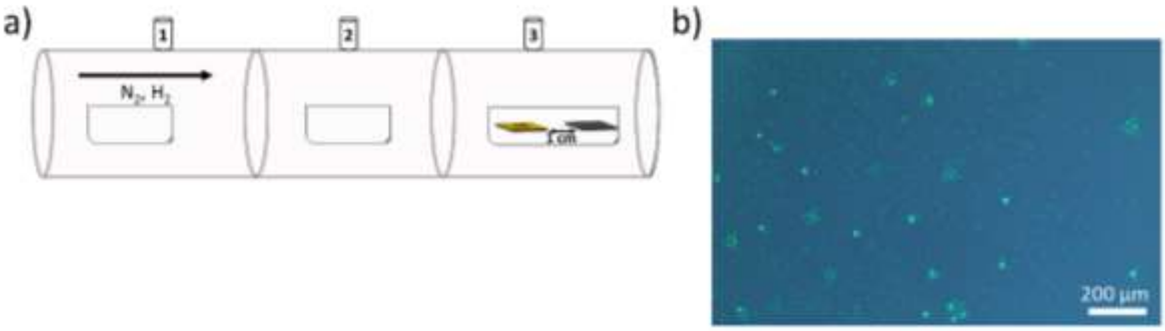

Figure S2: a) Diagram of the CVD setup used for both the pre-conversion and subsequent synthesis. b) Image of synthesized flakes from the same sample as shown in Figure 1-3 of the main text.

The following table lists a series of Raman parameters extracted following point spectra measurements from the same sample as shown in Figure 2 of the main text. Additionally, the value of the integrated intensity ratio between the L_E and A bands are also given, recorded from the same position. Average values for the spectra from the edge and center are respectively listed at the bottom of each column.

position	$A_{1g}-E_{1g}$ / cm^{-1}	$\frac{2LA(M)}{A_{1g}}$	$\frac{A_{1g}}{Si_{520}}$	$\frac{2LA(M)+E_{2g}}{A_{1g}}$	$\frac{LA(M)}{A_{1g}}$	Area($\frac{A_{1g}}{E_{2g}}$)	$\Gamma_{A_{1g}}$ / cm^{-1}	$\Gamma_{E_{1g}}$ / cm^{-1}	$\Gamma_{2LA(M)}$ / cm^{-1}	$\Gamma_{E_{2g}+2LA(M)}$ / cm^{-1}	$Pos_{A_{1g}}$ / cm^{-1}	$Pos_{E_{2g}}$ / cm^{-1}	$Pos_{2LA(M)}$ / cm^{-1}	Area($\frac{L_E}{A}$)
1 center	61.3	1.42	0.59	2.24	0.27	1.22	5.4	4.6	7.1	11.7	416.5	355.2	350.7	0.93
1 edge	61.8	1.42	0.74	2.62	0.09	1.01	4.9	4.0	7.0	11.1	416.5	354.7	350.2	1.67
2 center	61.4	1.73	0.71	3.00	0.12	1.09	5.2	3.8	7.4	11.2	417.1	355.7	351.3	2.32
2 edge	62.3	1.45	0.68	2.70	0.10	1.05	5.3	4.0	7.1	11.1	417.0	354.7	350.2	1.66
3 center	62.0	1.47	0.55	2.65	0.12	1.05	5.3	4.3	7.0	11.3	417.1	355.1	350.6	4.86
3 edge	62.4	1.42	0.69	2.66	0.09	1.06	5.4	4.1	7.0	11.1	417.3	354.9	350.4	4.01
4 center	62.8	1.29	0.74	2.43	0.08	1.08	5.2	4.2	6.9	11.1	417.6	354.8	350.4	2.67
4 edge	62.3	1.45	0.59	2.72	0.12	0.92	4.9	4.2	6.9	11.1	417.2	354.9	350.3	0.42
5 center	62.4	1.30	0.60	2.30	0.12	1.33	5.5	4.2	7.1	11.3	417.6	355.0	350.5	6.68
5 edge	62.2	1.65	0.75	3.12	0.10	0.88	5.3	4.1	7.0	11.0	417.2	355.0	350.5	2.19
6	62.0	1.31	0.52	2.34	0.16	1.25	5.5	4.2	7.1	11.4	417.2	355.2	350.7	3.42
7 center	63.2	1.11	0.47	1.94	0.17	1.15	4.6	4.8	7.6	12.4	417.2	354.0	349.2	0.00
7 edge	62.8	1.15	0.52	1.91	0.23	1.38	4.5	4.2	7.7	11.9	417.4	354.6	350.0	0.00
8	61.9	1.96	0.92	3.40	0.11	1.05	5.1	3.4	7.2	10.6	417.4	355.5	351.1	1.67
9 center	62.1	1.46	0.46	2.57	0.13	1.18	5.4	4.1	7.3	11.4	417.2	355.1	350.7	1.82
9 edge	62.2	1.52	0.52	2.88	0.14	0.95	5.2	4.0	6.9	10.9	417.2	354.9	350.3	1.69
10 edge	62.4	1.49	0.63	2.78	0.10	0.96	5.3	4.3	6.9	11.2	417.3	354.9	350.4	4.25
Center average	62.1 ± 0.6	1.5 ± 0.2	0.6 ± 0.1	2.5 ± 0.4	0.1 ± 0.1	1.2 ± 0.1	5.2 ± 0.3	4.2 ± 0.4	7.2 ± 0.2	11.4 ± 0.5	417.2 ± 0.3	355.1 ± 0.4	350.6 ± 0.5	2.7 ± 1.9
Edge average	62.3 ± 0.3	1.4 ± 0.1	0.6 ± 0.1	2.7 ± 0.3	0.1 ± 0.0	1.0 ± 0.1	5.1 ± 0.3	4.1 ± 0.1	7.1 ± 0.2	11.2 ± 0.3	417.2 ± 0.3	354.8 ± 0.1	350.3 ± 0.1	2.0 ± 1.4

Table S1. Table of extracted Raman characteristics from different flakes on the sample listed alongside the corresponding integrated intensity ratio of the L_E and A bands.

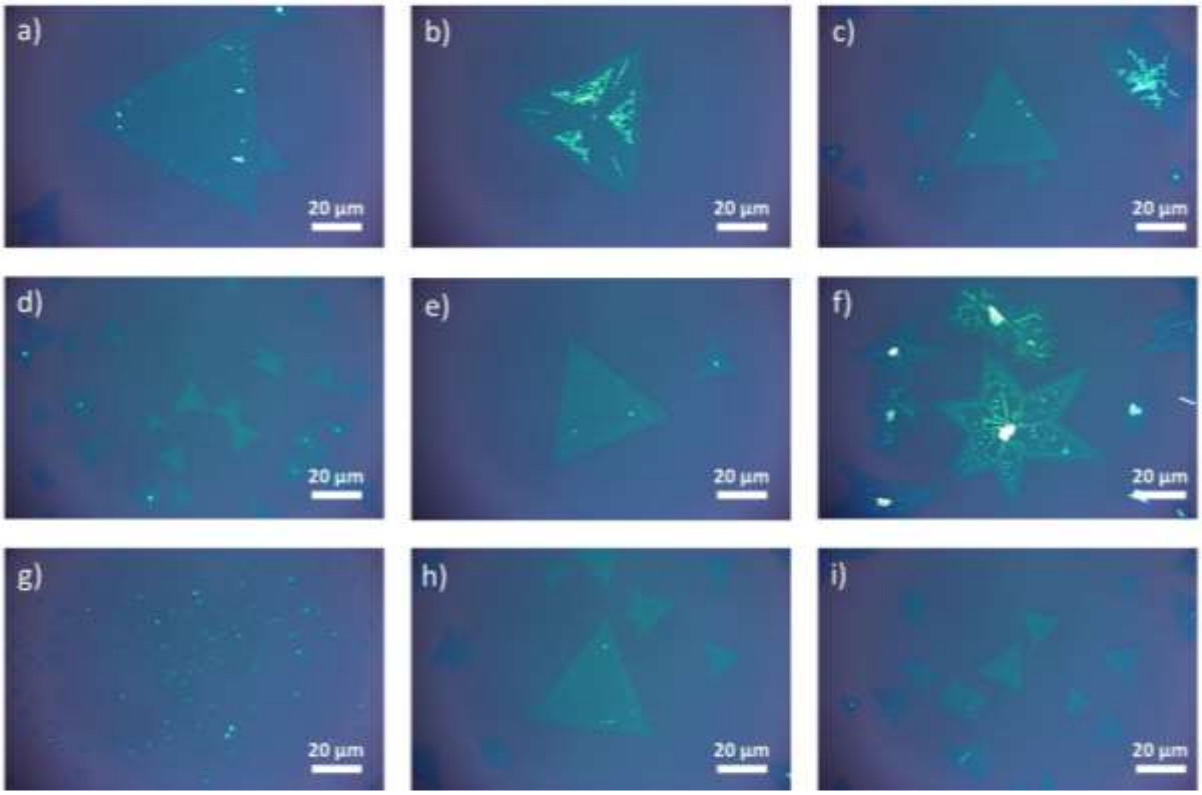

Figure S3: (a-i) Optical images from the flakes characterized by Raman and PL.

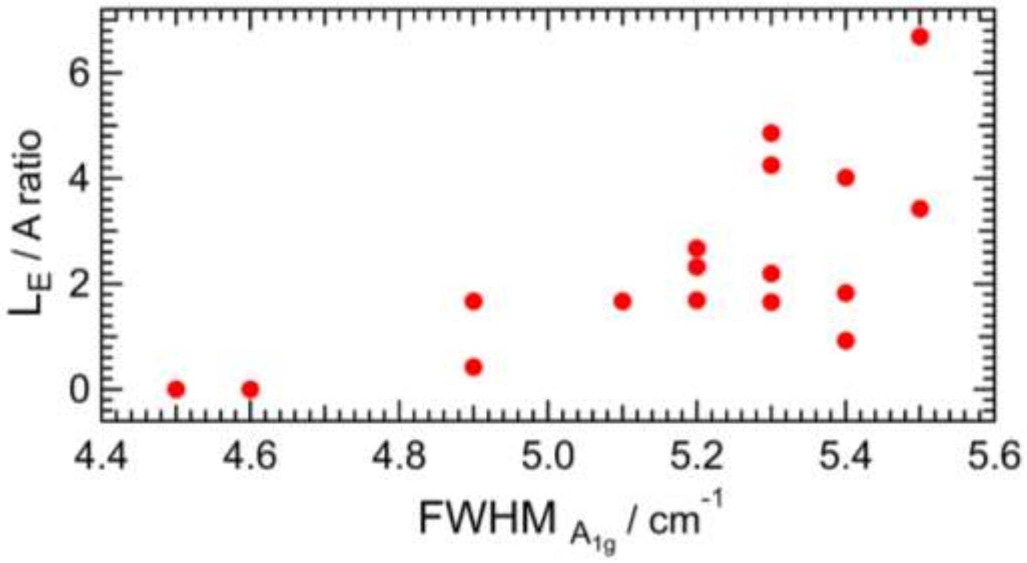

Figure S4. Graph showing the width of the A_{1g} band against the integrated intensity ratio of L_E and A bands.

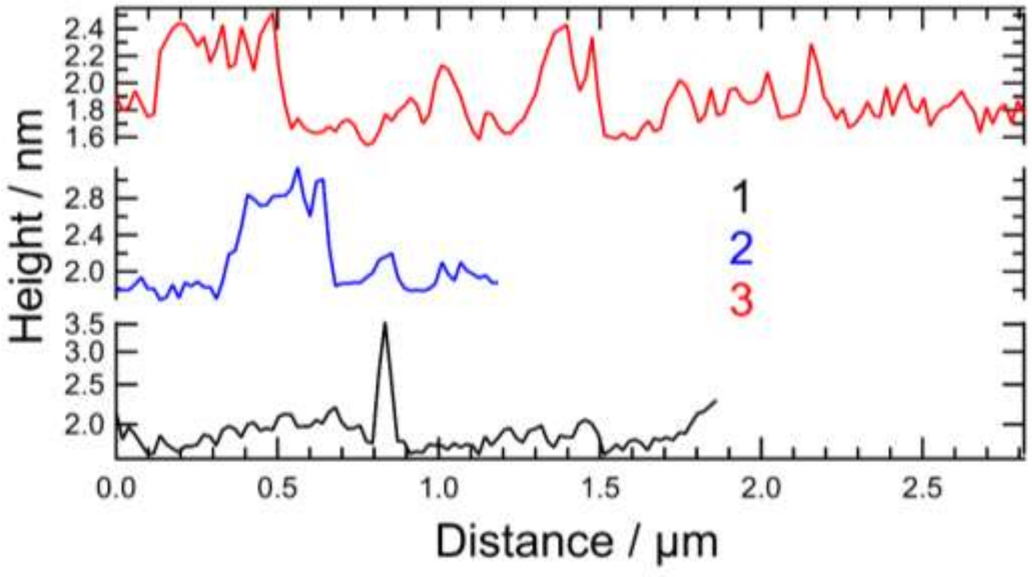

Figure S5: AFM height profiles derived from the line profiles in Figure 2d in the main text.

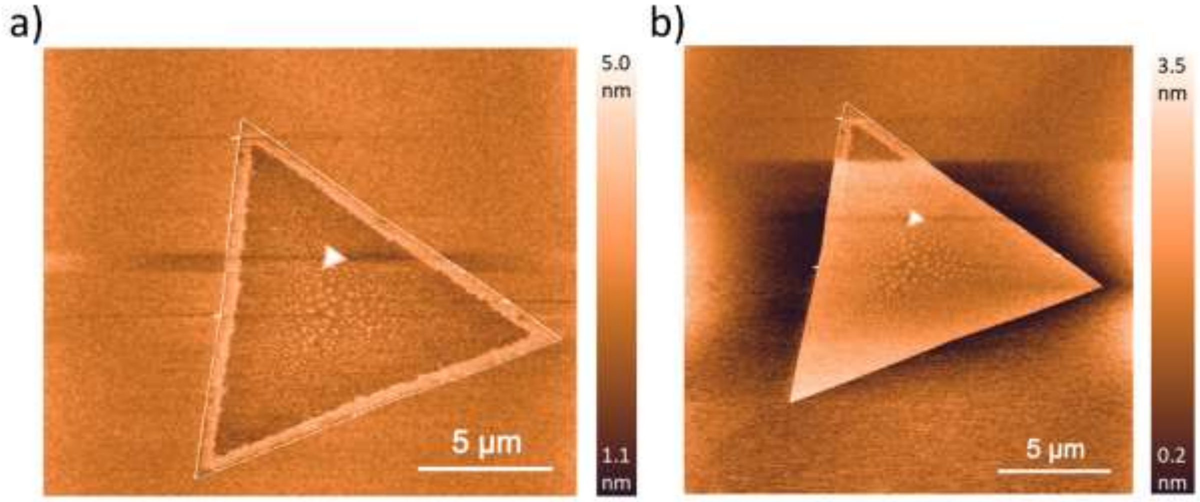

Figure S6. (a, b) Successive AFM height scans from the same WS2 flake.

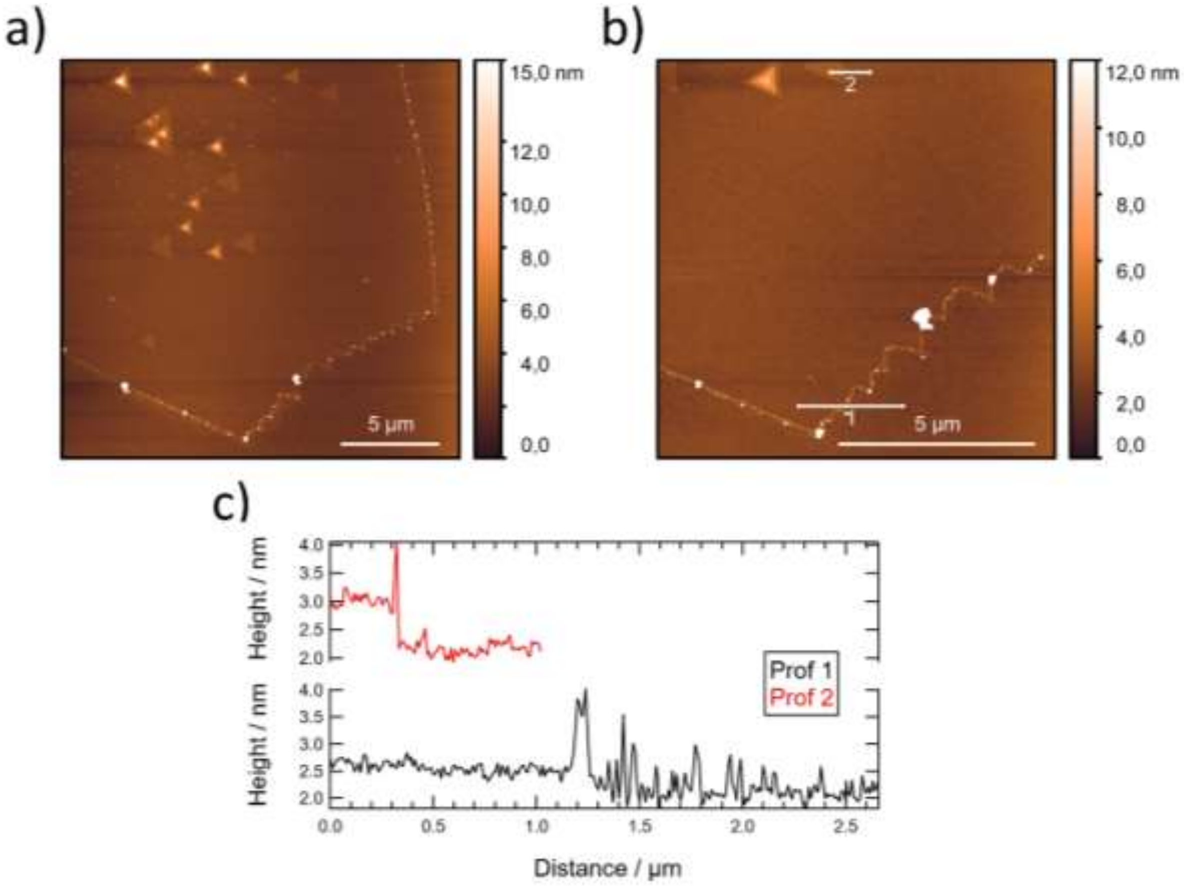

Figure S7. (a, b) AFM height images from a flake on the sample used in Figures 4, 5, and 6 of the main text. (c) AFM height profiles derived from the lines in (b).

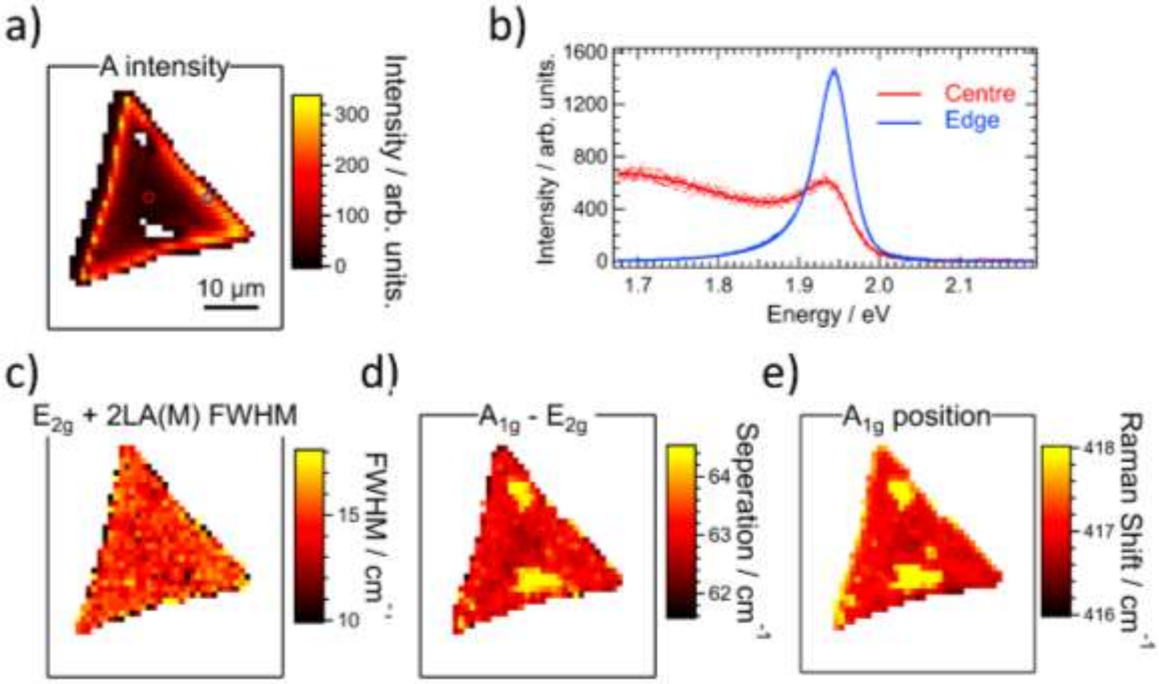

Figure S8. (a) Copy of Figure 4a from the main text, showing the A exciton integrated intensity with two positions marked by the red and blue circles in the center (red) and edge (blue) of the flake, respectively. (b) spectra and fitted curves from the center and edge of the flake, respectively, from the positions marked in (a). (c-e) Raman maps of the sum of the E_{2g} and 2LA(M) FWHM, the separation of the A_{1g} and E_{2g} bands, and the A_{1g} position, respectively.

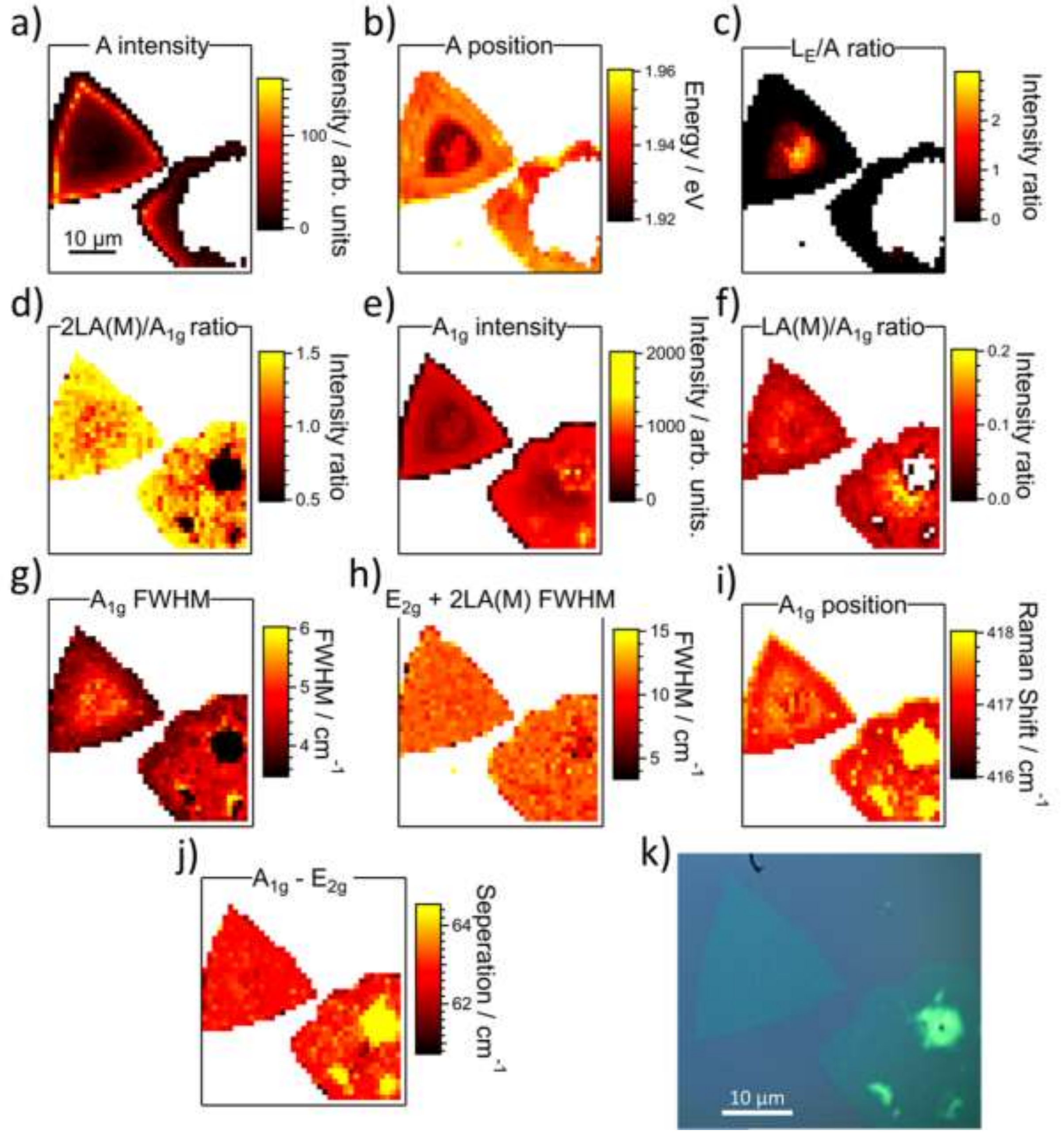

Figure S9: PL and Raman maps from a second area on the sample. (a-c) PL maps of the integrated intensity of the A exciton, band position of the A exciton, and integrated intensity ratio of the LE and A bands, respectively. (d-j) Raman maps of the 2LA(M) / A_{1g} intensity ratio, A_{1g} intensity, LA(M) / A1g intensity ratio, FWHM of the A_{1g} band, sum of the FWHM of the E_{2g} and 2LA(M) bands, A_{1g} position, and separation of the A_{1g} and E_{2g} bands, respectively. (k) optical image of the mapped area.

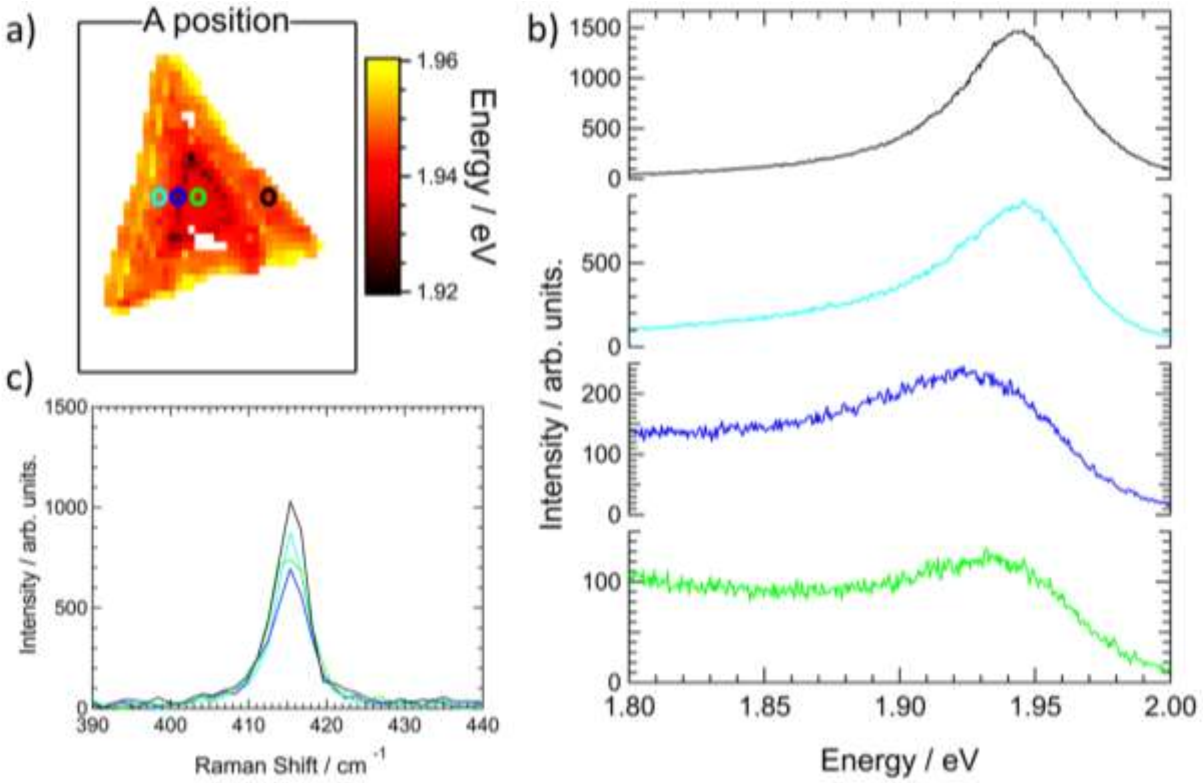

Figure S10: a) Copy of Figure 4b from the main text, showing the positions from which 4 spectra have been extracted. b, c) PL and Raman spectra extracted from the corresponding position in a). The Raman spectra are focused on the A_{1g} band only.

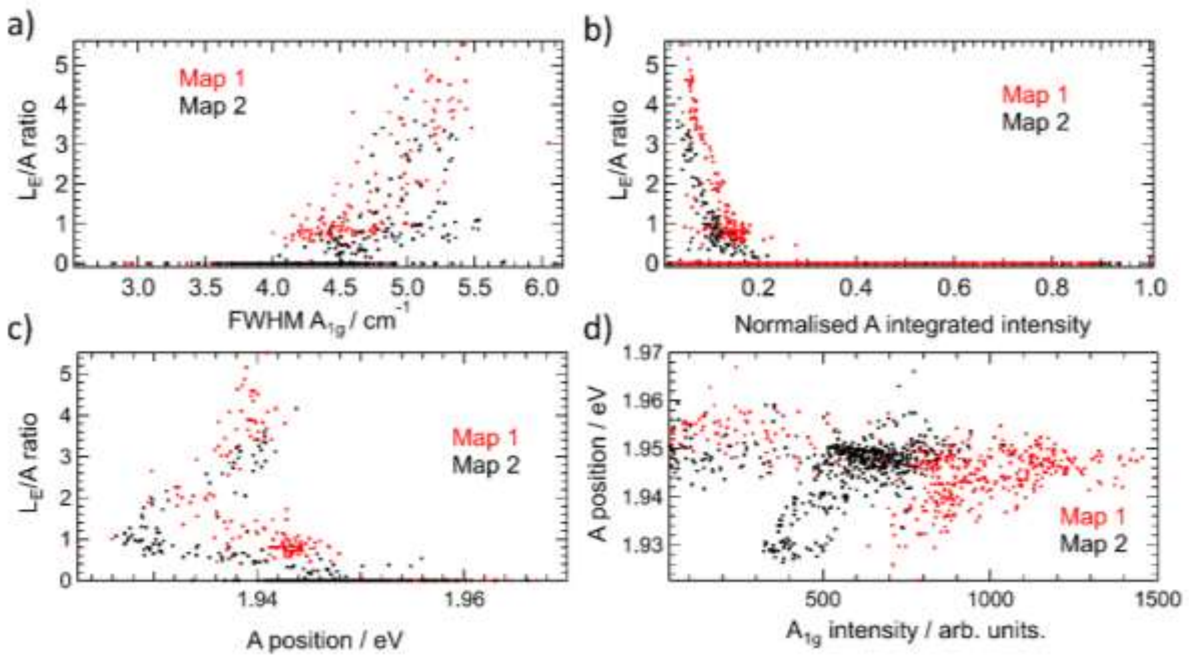

Figure S11. (a) Graph showing the FWHM of the Raman A_{1g} band against L_E / A integrated intensity PL ratio (b) corresponding graph showing the normalized A exciton integrated intensity against the L_e / A integrated intensity ratio. c) graph showing the A exciton position against L_e / A integrated intensity ratio. d) graph showing the A_{1g} intensity against A exciton position. All data points have been extracted from the maps shown in figures 4 and S9.

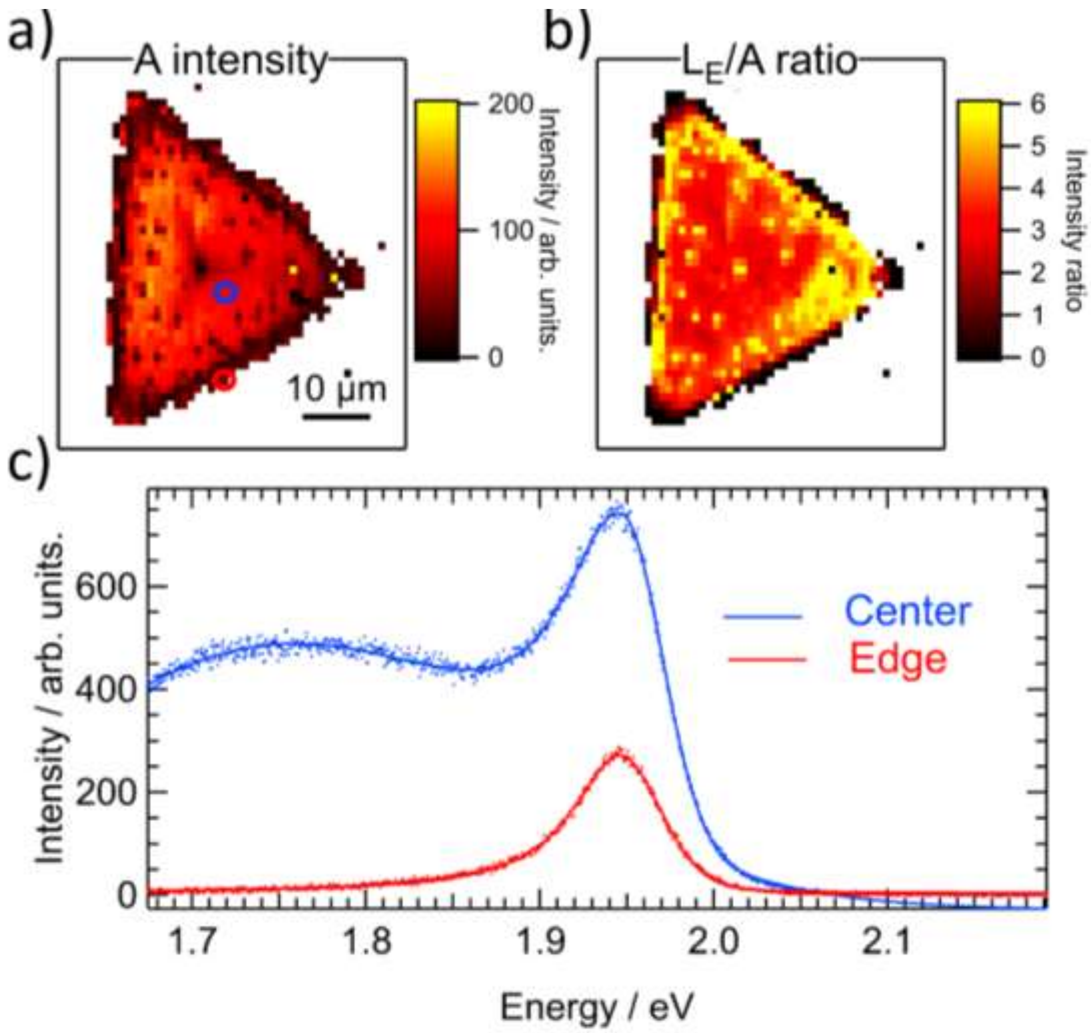

Figure S12. (a) Map of the exciton integrated intensity from a sample synthesized with 25 mg of sulfur added to zone 2 of the CVD chamber. (b) Corresponding map of the integrated intensity ratio of the L_E / A bands. (c) spectra along with multipeak fits from the positions marked in (a) from the center and edge, respectively.

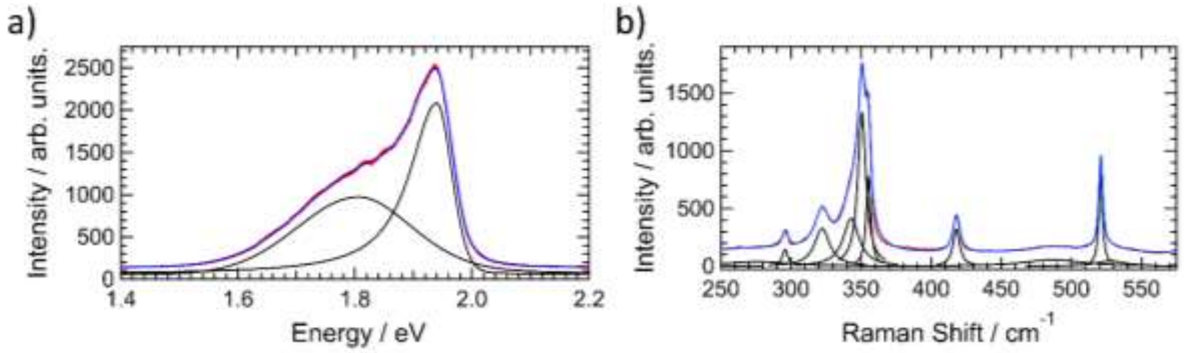

Figure S13. (a, b) PL (a) and Raman (b) spectra recorded from the synthesized WS_2 flakes recorded with 532 nm excitation. In both cases, raw data are given in red, and curves derived from multi-peak fitting given in blue. Individual bands are displayed in black.

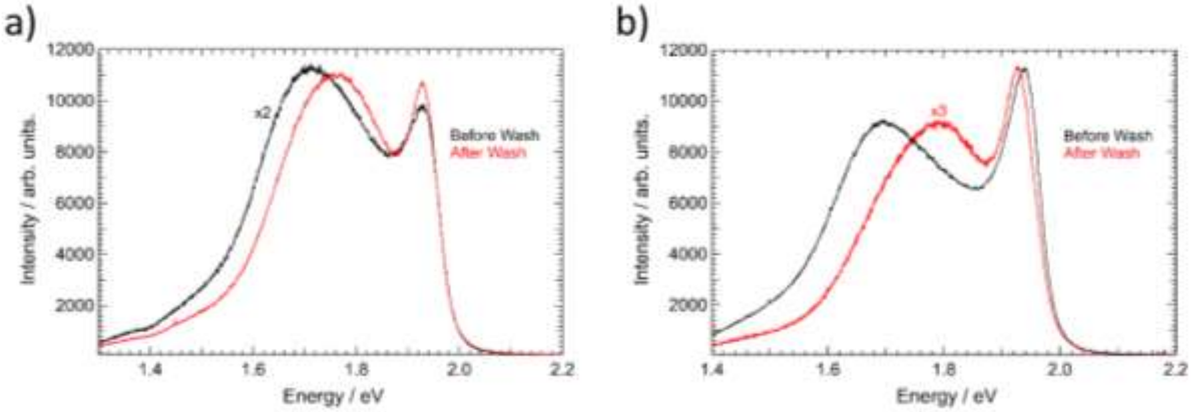

Figure S14. (a, b) PL spectra recorded from two positions before and after mild washing in milli-q water and acetone.
